# METTL3-dependent m^6^A modification of *PSEN1* mRNA regulates craniofacial development through the Wnt/β-catenin signaling pathway

**DOI:** 10.1038/s41419-024-06606-9

**Published:** 2024-03-20

**Authors:** Lan Ma, Xi Zhou, Siyue Yao, Xinyu Zhang, Ji Mao, Barbara Vona, Liwen Fan, Shu Lou, Dandan Li, Lin Wang, Yongchu Pan

**Affiliations:** 1 Jiangsu Province Key Laboratory of Oral Diseases, Nanjing Medical University, Nanjing, China; 2 Department of Orthodontics, The Affiliated Stomatology Hospital of Nanjing Medical University, Nanjing, China; 3 The Affiliated Stomatology Hospital of Suzhou Vocational Health College, Suzhou, China; 4 Institute of Human Genetics, University Medical Center Göttingen, Göttingen, Germany; 5 Institute for Auditory Neuroscience and Inner Ear Lab, University Medical Center Göttingen, Göttingen, Germany; 6 Jiangsu Province Engineering Research Center of Stomatological Translational Medicine, Nanjing Medical University, Nanjing, China

**Keywords:** Developmental biology, Epigenetics

## Abstract

Craniofacial malformations, often associated with syndromes, are prevalent birth defects. Emerging evidence underscores the importance of m^6^A modifications in various bioprocesses such as stem cell differentiation, tissue development, and tumorigenesis. Here, in vivo, experiments with zebrafish models revealed that *mettl3*-knockdown embryos at 144 h postfertilization exhibited aberrant craniofacial features, including altered mouth opening, jaw dimensions, ethmoid plate, tooth formation and hypoactive behavior. Similarly, low METTL3 expression inhibited the proliferation and migration of BMSCs, HEPM cells, and DPSCs. Loss of METTL3 led to reduced mRNA m^6^A methylation and PSEN1 expression, impacting craniofacial phenotypes. Co-injection of *mettl3* or *psen1* mRNA rescued the level of Sox10 fusion protein, promoted voluntary movement, and mitigated abnormal craniofacial phenotypes induced by *mettl3* knockdown in zebrafish. Mechanistically, YTHDF1 enhanced the mRNA stability of m^6^A-modified *PSEN1*, while decreased METTL3-mediated m^6^A methylation hindered β-catenin binding to PSEN1, suppressing Wnt/β-catenin signaling. Pharmacological activation of the Wnt/β-catenin pathway partially alleviated the phenotypes of *mettl3* morphant and reversed the decreases in cell proliferation and migration induced by METTL3 silencing. This study elucidates the pivotal role of METTL3 in craniofacial development via the METTL3/YTHDF1/PSEN1/β-catenin signaling axis.

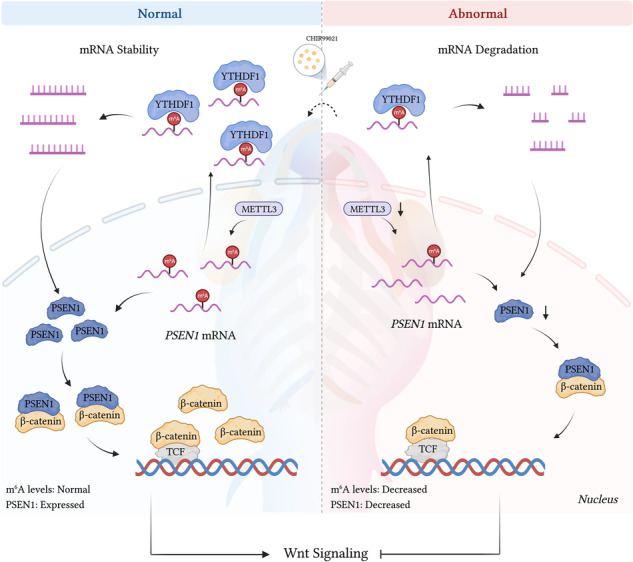

## Introduction

Craniofacial malformations are prevalent birth defects that often manifest as part of syndromes [1, 2]. These defects encompass a diverse array of phenotypes, such as cleft lip with or without cleft palate, cleft palate alone, craniosynostosis, craniofacial microsomia, and malocclusion [3–7]. The characterization of genetic and epigenetic factors in patients with dysmorphologies and animal models has illuminated the underlying etiological mechanisms contributing to craniofacial developmental abnormalities.

*N*^*6*^-methyladenosine (m^6^A), the most abundant internal modification in eukaryotic mRNAs, is critically important for various developmental events [8, 9]. Dynamic and reversible RNA methylation is orchestrated by writer proteins (methyltransferases), eraser proteins (demethylases) and reader proteins (RNA-binding proteins) [10, 11]. Methyltransferase-like 3 (METTL3), methyltransferase-like 14 (METTL14) and Wilms’ tumor 1-associated protein (WTAP) constitute the core of methyltransferase complex [12–14]. The stable depletion of m^6^A through *Mettl3* knockout in mice leads to embryonic lethality and activates the pErk and pAkt signaling pathways, facilitating pluripotency departure [15, 16]. Mettl14 has been implicated in the regulation of embryonic neural stem cell self-renewal and brain development via histone modifications [17]. Conversely, the fat mass and obesity–associated protein (FTO) and alkB homolog 5 (ALKBH5) serve as demethylases, reversing m^6^A modifications. Recent studies have revealed that FTO-mediated m^6^A demethylation of *LINE1* plays a regulatory role in shaping the chromatin state and gene expression in mouse oocytes and embryonic stem cells [18].

Accumulating evidence underscores the crucial role of m^6^A modifications in shaping RNA fate and functions, including mRNA stability, localization, splicing, transport, translation, microRNA processing, and RNA-protein interactions. Moreover, m^6^A modifications are critical for diverse bioprocesses, ranging from stem cell differentiation, tissue development, and sex determination to tumorigenesis [19–24]. Despite this understanding, the physiological role of m^6^A modifications in abnormal craniofacial development has not been fully elucidated.

In this study, we conducted in vivo and in vitro experiments using zebrafish and cell models to explore the biological function of m^6^A methylase METTL3 involved in embryonic craniofacial development. Furthermore, we observed a significant decrease in *psen1* expression upon *mettl3* knockdown. Mechanistically, Mettl3-mediated m^6^A modification appeared to regulate the level of Sox10, possibly by affecting *psen1* in a YTHDF1-dependent manner, thereby influencing Wnt/β-catenin signaling. Collectively, our findings provide compelling evidence for the crucial involvement of the METTL3/YTHDF1/PSEN1/β-catenin axis in vertebrate embryonic craniofacial developmental events.

## Results

### METTL3 is involved in craniofacial development

We investigated the potential relevance of m^6^A modifications to developmental processes and congenital diseases. To identify critical m^6^A writers in this context, we conducted a comprehensive literature search using PubMed, Google Scholar and Web of Science databases up to August 2023. Each m^6^A writer was assigned a score by summing the score for the studies included (1 if it was reported, 0 if it was not reported). Ultimately, 21 studies were identified, and among them, METTL3 emerged with the highest score for m^6^A modification, with a score exceeding 10 (Supplementary Fig. 1A, Supplementary Table 1). By analyzing spatial transcriptome data from C57BL/6 mouse embryos at E16.5, we observed that the expression of *Mettl3* was predominantly clustered in regions associated with jaw, tooth, and mucosal epithelium (Supplementary Fig. 1B). Furthermore, the spatiotemporal mapping of developmental trajectories during zebrafish embryogenesis at 12 h postfertilization (hpf) highlighted the expression of *mettl3* in neural crest clusters, a critical region that has been linked to craniofacial development (Supplementary Fig. 1C).

### Expression pattern of Mettl3 in early zebrafish development

Bioinformatics analysis revealed that *mettl3* encodes a 70 kDa protein with a conserved catalytic DPPW motif (D399-W402), implying potential methylation activity for zebrafish Mettl3 (Supplementary Fig. 2A). Notably, the amino acid residue D395 in the S-adenosylmethionine (SAM) binding motif is critical for methyltransferase (MTase) activity, and W398 has been implicated in π–π stacking with the methylated adenine base during substrate binding in *Homo sapiens* [25]. Remarkably, alignment analysis revealed that the residue D399 of Mettl3 in *Danio rerio* exhibited evolutionary conservation with D395. This analysis also highlighted a relatively high homology between zebrafish *mettl3* and human *METTL3* (similarity, 68.3%).

Next, we investigated the expression pattern of *mettl3* during various zebrafish embryonic stages. Early expression of *mettl3* in zebrafish embryos decreased from 12 to 24 hpf (Supplementary Fig. 2B). These data collectively suggest a potentially important role for Mettl3 in zebrafish craniofacial development.

### Mettl3 deficiency results in craniofacial abnormalities in zebrafish

**Fig. 1:**
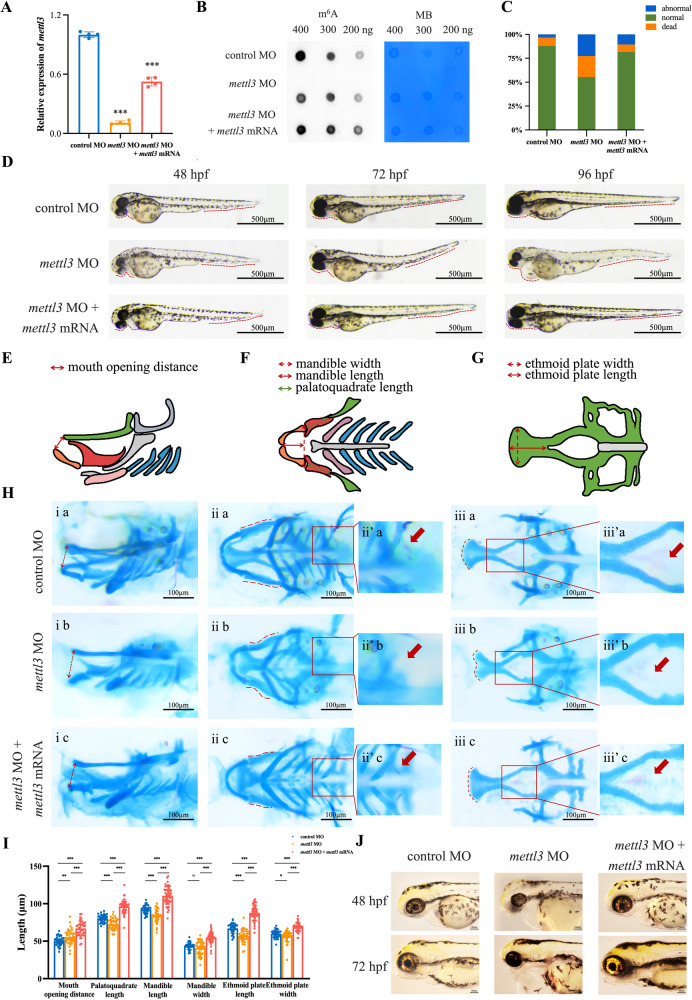
Phenotypes of the zebrafish. **A** The expression of *mettl3* in zebrafish embryos injected with control morpholino (MO), *mettl3* MO, or co-injection with *mettl3* MO and mRNA at 48 hpf. **B** Representative dot blot showing m^6^A levels in zebrafish embryos injected with control MO, *mettl3* MO, or co-injection with *mettl3* MO and mRNA. MB, methylene blue staining. **C** Statistical analysis of the number of dead, abnormal or normal embryos. **D** Lateral view of zebrafish larvae injected with control MO, *mettl3* MO, or co-injection with *mettl3* MO and mRNA that were imaged with transmitted light at 48, 72, and 96 hpf. **E**–**G** Schematic diagram of zebrafish craniofacial cartilage structures, including the distance of mouth opening, width and length of the mandible, length of the palatoquadrate, and width and length of the ethmoid plate, from lateral view and ventral views. **H** Zebrafish embryos at 144 hpf were stained with alcian blue and alizarin red to observe craniofacial structures. The red arrow shows the development of tooth and pharyngeal in zebrafish embryos. **I** Scatter histogram showing the length of the palatoquadrate, Meckel’s cartilage, and the ethmoid plate; the width of Meckel’s cartilage and the ethmoid plate; and the distance of mouth opening in zebrafish embryos injected with control MO, *mettl3* MO, or co-injection with *mettl3* MO and mRNA (each group, *n* = 100). **J** Iridophores at 48 and 72 hpf in zebrafish embryos injected with control MO, *mettl3* MO, or co-injection with *mettl3* MO and mRNA. Results were presented as mean ± SD of three independent experiments. *P < 0.05, **P < 0.01 or ***P < 0.001 indicates a significant difference between the groups.

To investigate the function of *mettl3* in craniofacial development, we generated a zebrafish model in which *mettl3* was specifically knocked down with morpholinos (MO). Injection of 8 ng of *mettl3* MO led to reduced *mettl3* and m^6^A levels, subsequently affecting embryo survival and increasing the incidence of malformations (Fig. 1A–C). Similarly, zebrafish embryos injected with *mettl3* MO exhibited a significant reduction in body length, heart-associated edema, and craniofacial abnormalities when compared to the controls (Fig. 1D). Notably, co-injection of *mettl3* mRNA containing *mettl3* MO target sequence effectively rescued both *mettl3* and m^6^A levels, alongside craniofacial phenotypes (Fig. 1A–D).

Building on these findings, we performed alcian blue and alizarin red staining to explore craniofacial defects in *mettl3* morphants. This encompassed assessing parameters such as the mouth opening distance, palatoquadrate and mandible length, as well as ethmoid plate length and mandible and ethmoid plate width (Fig. 1E–G). Compared to control morphants, the *mettl3*-knockdown embryos at 144 hpf exhibited increased mouth opening distance and decreased palatoquadrate and mandible lengths, analogous to the upper and the lower jaw structures, respectively (Fig. 1H, I). Additionally, *mettl3* morphants displayed a shorter ethmoid plate length and width, analogous to the human palate (Fig. 1H, I). Interestingly, significant changes in tooth formation and a decrease in iridophores were detected in the *mettl3* morphants (Fig. 1H–J). Importantly, all these defects were also significantly reversed by the expression of *mettl3* mRNA (Fig. 1).

Furthermore, we explored the levels of Sox2 and Sox3, required for early embryonic craniofacial differentiation in zebrafish. Notably, no significant differences in Sox2 or Sox3 levels were observed in the *mettl3*-knockdown zebrafish embryos at 72 or 96 hpf (Supplementary Fig. 2C). Collectively, our data suggest that Mettl3 may exert a critical role in embryonic craniofacial developmental processes, possibly through its methylation activity in zebrafish.

### Genetic associations in *METTL3* related to craniofacial development-linked diseases

Next, we evaluated the genetic associations of *METTL3* with craniofacial development-related diseases. In total, we identified four, six, and six independent genetic variants in *METTL3* associated with skeletal sagittal malocclusion, hard palate cleft, and tooth development and eruption, respectively (r^2^ < 0.6, *P* < 5 × 10^−2^) (Supplementary Table 2). To provide insights into the underlying molecular mechanisms, we highlight the variants with a relatively large effect. The rs1263800 and rs1263790 variants were associated with an increased risk for both skeletal sagittal malocclusion and tooth development and eruption. We embarked on a thorough extrapolation of the comprehensive catalog detailing genetic influences on gene expression, wherein the rs1263800 variant demonstrated a significant association with *METTL3* expression in skeletal muscle (Supplementary Fig. 3A, B). Similarly, the risk allele of rs1263790 displayed an association with reduced expression of its corresponding gene *METTL3* based on analysis of eQTLGen Consortium data (z-score = −6.806, *P* = 1.00 × 10^−11^).

### Impact of low METTL3 expression on cell proliferation and migration

**Fig. 2:**
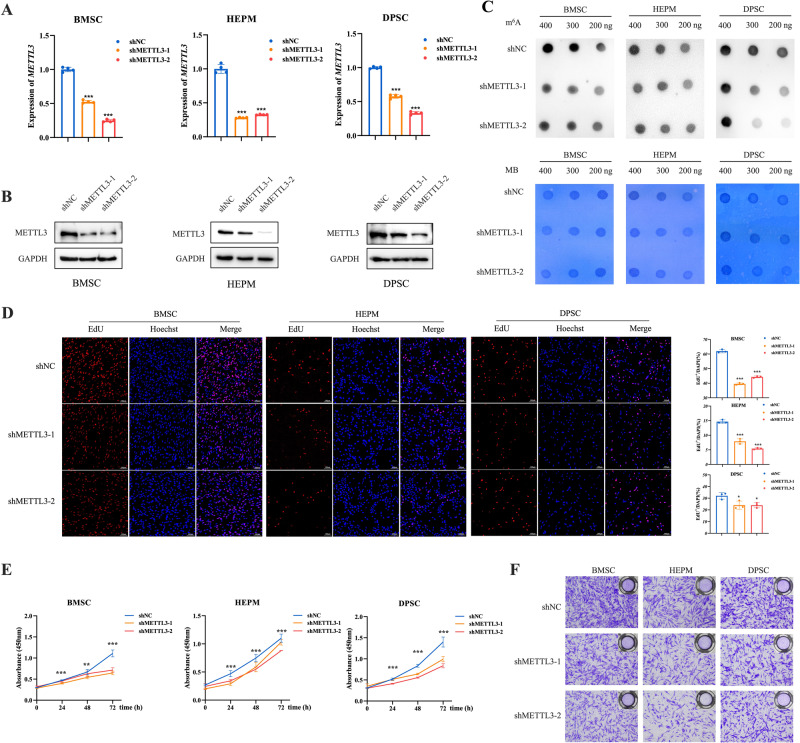
METTL3 knockdown significantly suppresses cell proliferation and migration in vitro. **A**, **B** The efficiency of METTL3 knockdown in BMSCs, HEPM cells, and DPSCs. The expression of METTL3 was verified at both the mRNA and protein levels. **C** Representative dot blot showing the m^6^A levels in cells with METTL3 knockdown and control groups. MB, methylene blue staining. **D**, **E** Low METTL3 expression significantly reduced the proliferation rate of BMSCs, HEPM cells, and DPSCs. **F** Low METTL3 expression significantly reduced the migration ability of BMSCs, HEPM cells, and DPSCs. Results were presented as mean ± SD of three independent experiments. *P < 0.05, **P < 0.01 or ***P < 0.001 indicates a significant difference between the designated groups.

To further elucidate the functional roles of METTL3 in vitro, we selected human bone marrow mesenchymal stem cells (BMSCs), human embryonic palatal mesenchymal (HEPM) cells and human dental pulp stromal cells (DPSCs), each of which corresponds to distinct craniofacial phenotypes observed in zebrafish embryos or has been linked to craniofacial development-linked diseases. A negative control shRNA construct and two *METTL3*-specific shRNA constructs were designed. Subsequently, qRT-PCR and western blot assays were performed to confirm the successful knockdown of METTL3 in the cells (Fig. 2A, B). As expected, METTL3 knockdown significantly reduced the m^6^A levels (Fig. 2C). Knockdown of METTL3 distinctly inhibited the proliferation rate of the three cell types (Fig. 2D, E). Moreover, we found that decreased METTL3 expression correlated with a notable decrease in cell migration in transwell assays (Fig. 2F). Collectively, these data suggested that low expression of METTL3 could effectively suppress the proliferation and migration of BMSCs, HEPM cells and DPSCs, highlighting the potential regulatory roles of METTL3 in craniofacial development.

### Identification of METTL3 targets by high-throughput RNA-seq and m^6^A-seq

**Fig. 3:**
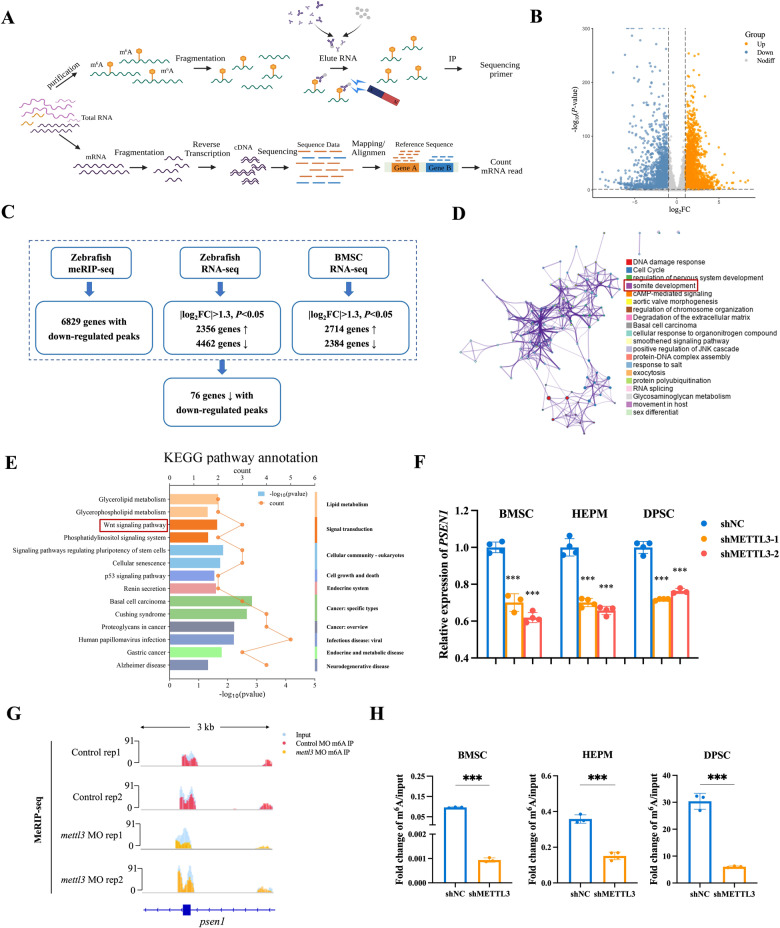
Identification of METTL3 targets via MeRIP-seq and RNA-seq. **A** Schematic diagram depicting the protocols used for MeRIP-seq and RNA-seq. **B** Volcano plot of differentially expressed genes between METTL3 knockdown and control BMSCs. **C** Flow chart showing the shared downregulated genes with hypomethylated m^6^A peaks. **D** Gene enrichment analysis performed via the Metascape database. **E** KEGG pathway enrichment analysis shows major signaling pathways in METTL3-knockdown BMSCs compared to control BMSCs. **F** The expression level of *PSEN1* in BMSCs, HEPM cells, and DPSCs in the METTL3-knockdown and control groups. **G** The m^6^A abundances of *psen1* transcript in *mettl3*-knockdown zebrafish embryos compared to control embryos. **H** Validation of m^6^A modification in METTL3-knockdown and control cells using MeRIP-qPCR. Results were presented as mean ± SD of three independent experiments. *P < 0.05, **P < 0.01 or ***P < 0.001 indicates a significant difference between the groups.

To explore the mechanistic regulations of METTL3-induced m^6^A modification, we initially retrieved the MeRIP-seq and RNA-seq data from *mettl3* MO zebrafish embryos [26] and performed RNA-seq analysis of stable METTL3 knockdown or control BMSCs (Fig. 3A). The MeRIP-seq analysis revealed hypomethylation of 6829 genes within *mettl3*-deficient zebrafish embryos. Similarly, when comparing *mettl3* MO zebrafish embryos and controls, 6818 genes were found to be differentially expressed (2356 upregulated and 4462 downregulated; |log_2_FC | > 1.3, *P* < 0.05). Furthermore, METTL3 knockdown in BMSCs resulted in the differential expression of 5098 genes (2714 upregulated and 2384 downregulated; |log_2_FC | > 1.3, *P* < 0.05) (Fig. 3B). This analysis ultimately revealed 76 overlapping downregulated genes accompanied by hypomethylated m^6^A peaks (Fig. 3C). Metascape analysis showed that the 76 genes were primarily associated with somite development, highlighting the crucial role of m^6^A modification in embryonic development (Fig. 3D). Kyoto Encyclopedia of Genes and Genomes (KEGG) analysis revealed that the major affected pathways encompassed Wnt signaling pathway and p53 signaling pathway (Fig. 3E). Previous studies found that Wnt signaling is critical to early embryo development and the biology of stem cells and progenitors [27, 28], thus we focused on the Wnt signaling pathway.

To validate these findings, qRT‒PCR assays further confirmed the reduction in the expression of only *PSEN1*, a component of the Wnt signaling pathway, in METTL3-knockdown cells (Fig. 3F and Supplementary Fig. 4A, B). Similarly, *psen1* mRNA levels were significantly lower in *mettl3*-knockdown zebrafish embryos than in control zebrafish embryos (Supplementary Fig. 4C). scRNA-seq analysis of expression data from bone marrow in the Tabula Sapiens revealed that *PSEN1* was expressed mainly in hematopoietic stem cells, closely related to *METTL3* expression (Supplementary Fig. 4D). MeRIP-seq further unveiled a diminished m^6^A peak in *PSEN1* within *mettl3*-deficient zebrafish embryos (Fig. 3G). Consistent with these findings, MeRIP-qPCR confirmed that METTL3 silencing decreased the m^6^A levels in *PSEN1* (Fig. 3H).

### *PSEN1* modulation by METTL3-mediated m^6^A RNA methylation

**Fig. 4:**
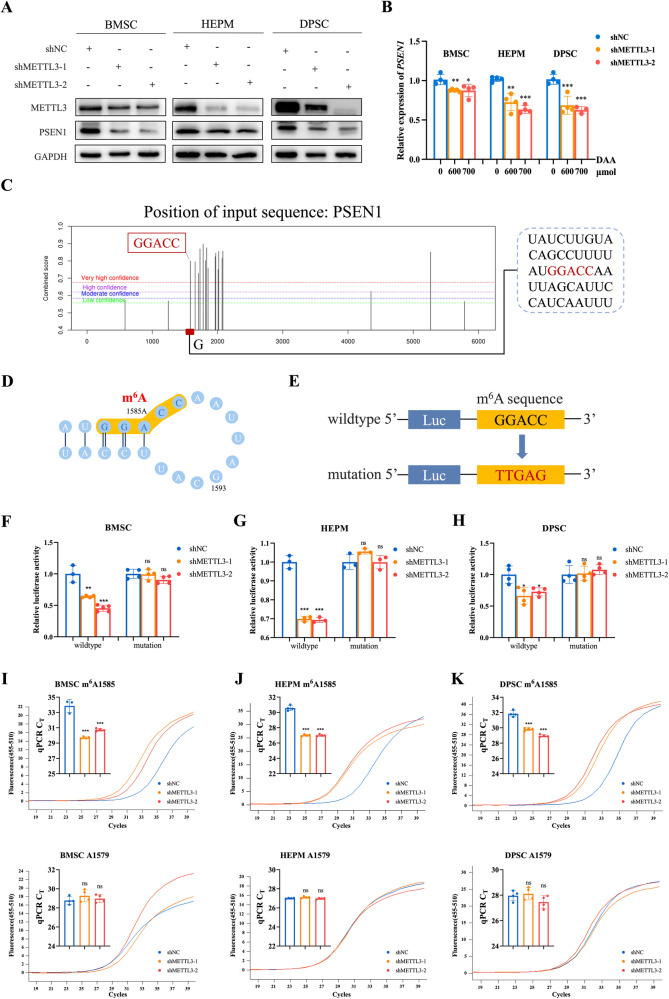
PSEN1 is modulated by METTL3-mediated m^6^A RNA methylation. **A** The protein level of PSEN1 in BMSCs, HEPM cells and DPSCs with METTL3-knockdown and control groups. **B** The relative expression of *PSEN1* detected at the RNA level after treatment with DAA in at various concentrations (0 μmol, 600 μmol and 700 μmol). **C**, **D** Potential m^6^A sites in full-length *PSEN1* gene predicted using SRAMP. **E** The wild-type or mutant m^6^A consensus sequence fused with the firefly luciferase reporter. **F**–**H** The transcription levels of wild-type and mutant *PSEN1* in BMSCs, HEPM cells and DPSCs. **I**–**K** The m^6^A methylation level of the *PSEN1* at specific modification site (m^6^A1585) and control site (A1579) using SELECT in control and METTL3 knockdown cells. Results were presented as mean ± SD of three independent experiments. *P < 0.05, **P < 0.01 or ***P < 0.001 indicates a significant difference between the groups.

Furthermore, we assessed the PSEN1 protein levels in METTL3 knockdown cells, which exhibited significant downregulation (Fig. 4A). To confirm our hypothesis, we employed the methylation inhibitor 3-deazaadenosine (DAA). Remarkably, treatment with DAA at varying concentrations led to a considerable reduction in the *PSEN1* levels (Fig. 4B). To determine whether the regulatory effect of METTL3 on PSEN1 is dependent on the methylation of its mRNA transcript targets, we predicted potential m^6^A sites in the full-length *PSEN1* gene via SRAMP (Fig. 4C, D) and constructed luciferase reporter plasmids with the wild-type or mutant m^6^A consensus sequence (GGACC to TTGAG) in *PSEN1* (Fig. 4E). Luciferase assays showed that only wild-type *PSEN1* significantly suppressed the transcription of *PSEN1* in stable METTL3 knockdown cells (Fig. 4F–H). To further verify the m^6^A modification sites at the *PSEN1* transcript, the single-base elongation- and ligation-based qPCR amplification method SELECT was used and the probe pairs targeting the highly confident m^6^A1585 site and A1579 control site at *PSEN1* transcript respectively were designed. We observed that m^6^A1585 targeted site revealed significantly decreased m^6^A levels after METTL3 was knocked down (Fig. 4I–K).

**Fig. 5:**
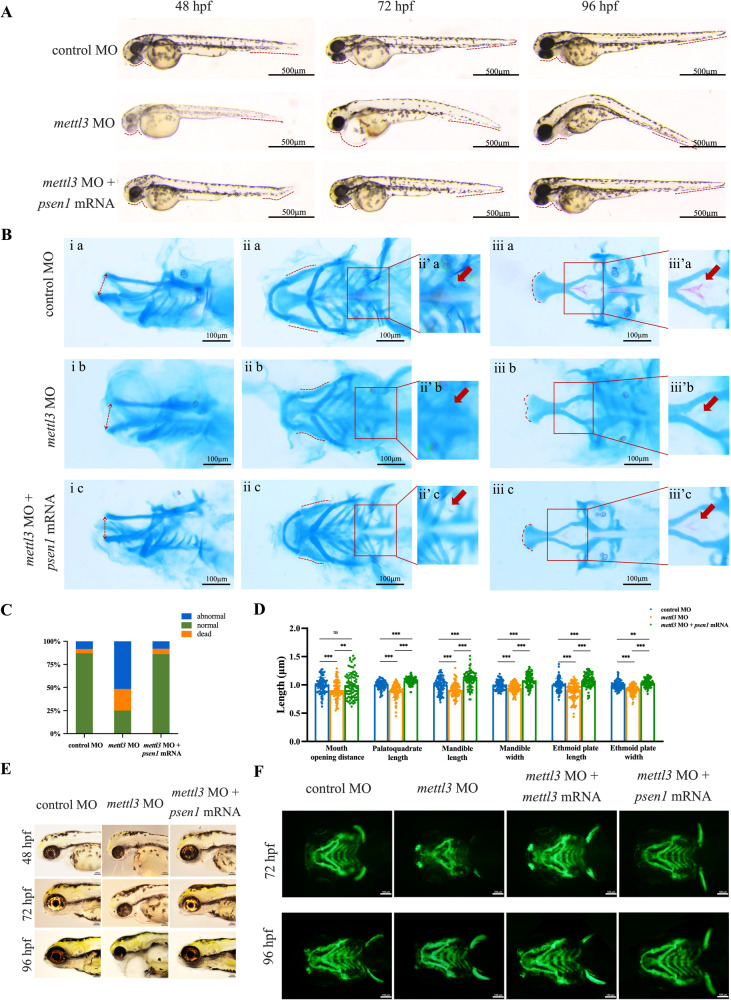
*psen1* rescues the level of Sox10 associated with migrating zebrafish neural crest cells and abnormal craniofacial phenotypes. **A**, **B** Co-injection of *psen1* mRNA rescued the abnormal craniofacial phenotypes in *mettl3*-knockdown embryos. **C** Statistical analysis of the number of dead, abnormal, or normal embryos. **D** Scatter histogram showing the length of the palatoquadrate, Meckel’s cartilage, and the ethmoid plate; the width of Meckel’s cartilage and the ethmoid plate; and the distance of the mouth opening in zebrafish embryos injected with control MO, *mettl3* MO, or co-injection with *mettl3* MO and *psen1* mRNA (each group, *n* = 100). **E** Iridophores at 48, 72, and 96 hpf in zebrafish embryos injected with control MO, *mettl3* MO, or co-injection with *mettl3* MO and *psen1* mRNA. **F** Tg(*sox10*: eGFP) transgenic zebrafish embryos expressing green fluorescent protein (GFP) were used to explore the effects of *mettl3* and *psen1* during zebrafish embryogenesis. Results were presented as mean ± SD of three independent experiments. *P < 0.05, **P < 0.01 or ***P < 0.001 indicates a significant difference between the groups.

Next, we determined whether METTL3 regulated cell proliferation and migration via *PSEN1* and found that *PSEN1* overexpression partially reversed the decreased proliferation and migration in METTL3-silenced cells (Supplementary Fig. 5). We used bioinformatics to analyze the spatial transcriptomics data of zebrafish embryogenesis at 18 hpf. *psen1* and *mettl3* exhibited similar expression patterns, particularly in the neural crest (Supplementary Fig. 6A). Additionally, early *psen1* expression was decreased in zebrafish embryos from 12 hpf to 24 hpf, which was closely correlated with *mettl3* expression (Supplementary Fig. 6B, C). To assess the involvement of *psen1* in the regulation of craniofacial abnormalities by *mettl3* during zebrafish embryogenesis, we designed a rescue experiment (Supplementary Fig. 6D). Co-injection of *psen1* mRNA significantly rescued abnormal craniofacial phenotypes in *mettl3* knockdown embryos (Fig. 5A–E). Moreover, considering that neural crest cells (NCCs) play an important role in zebrafish craniofacial development and that *sox10* is expressed in migrating zebrafish NCCs [29], we used *Tg*(*sox10:eGFP*) transgenic zebrafish embryos expressing green fluorescent protein (GFP) to study the effects of *mettl3* and *psen1* during zebrafish embryogenesis. Consistent with our prediction, the levels of both Sox10 fused to the GFP protein and Sox10 were weak in the *mettl3* morphants (Fig. 5F and Supplementary Fig. 6E). Importantly, co-injection of *mettl3* or *psen1* mRNA rescued the level of Sox10 fusion protein (Fig. 5F and Supplementary Fig. 6F). Taken together, our results suggest that Mettl3-mediated m^6^A modification likely regulates the level of Sox10, potentially via its effect on *psen1*, thereby influencing craniofacial developmental events in vertebrate embryogenesis.

### YTHDF1, an m^6^A reader protein, maintains *PSEN1* mRNA stability

**Fig. 6:**
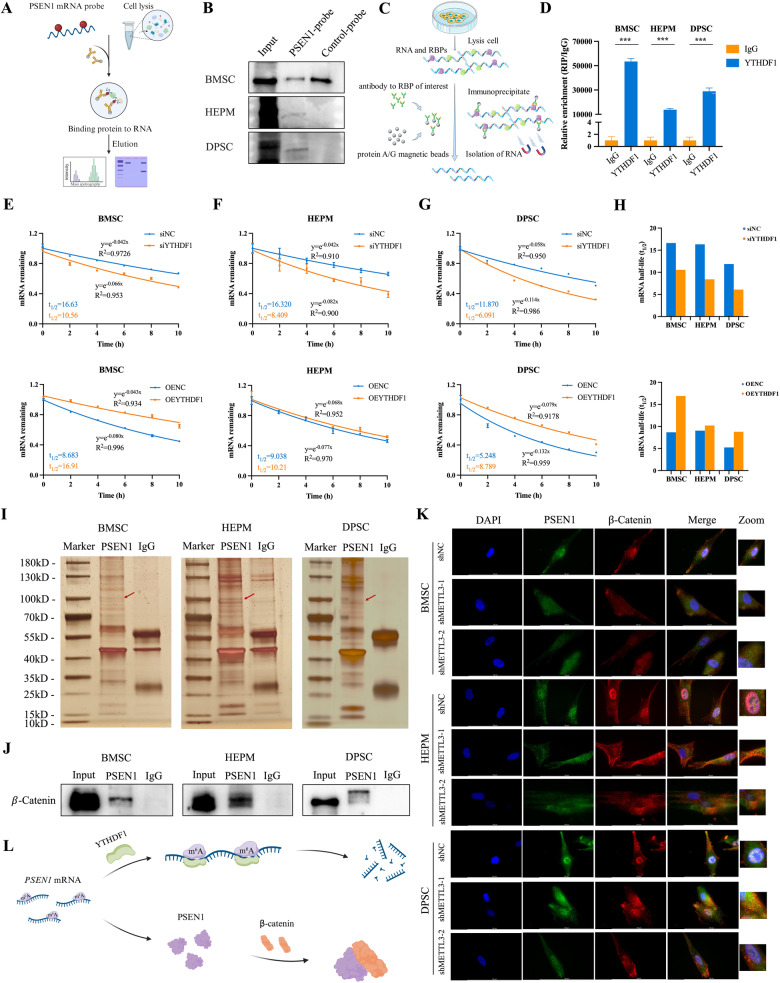
PSEN1 is specifically recognized by YTHDF1 and directly interacts with β-catenin. **A** Schematic of the design for the RNA pull-down assay. **B** Immunoblotting of YTHDF1 after the RNA pull-down assay with cell lysate, biotinylated-*PSEN1*, and biotinylated-control in the cells. **C** Schematic of the design for the RNA immunoprecipitation (RIP) assay. **D** RIP assay to determine the enrichment of *PSEN1* in cells incubated with anti-YTHDF1 antibody. **E**–**H** Cells were transiently transfected with control, siYTHDF1, empty vector, or OEYTHDF1, respectively. The half-life (t_1/2_) of the *PSEN1* mRNA was measured. **I** Silver staining revealed PSEN1-bound proteins in BMSCs, HEPM cells and DPSCs. **J** Interaction between β-catenin and PSEN1 determined by co-IP followed by western blot analysis. **K** Representative image showing the enrichment of PSEN1 and β-catenin after METTL3 knockdown by immunostaining analyses in BMSCs, HEPM cells, and DPSCs. **L** Schematic diagram showing the mechanism of PSEN1. Results were presented as mean ± SD of three independent experiments. ***P < 0.001 indicates a significant difference between the groups.

Previous studies have indicated the important roles of m^6^A reader proteins in the regulation of RNA modifications [10]. To identify the m^6^A reader that recognizes and binds *PSEN1* for methylation, an RNA pull-down assay employing biotin-labeled *PSEN1* mRNA from BMSCs, HEPM cells, and DPSCs was conducted (Fig. 6A). Mass spectrometry (MS) analysis of the corresponding bands revealed that YTHDF1 emerged as the prominent m^6^A reader protein in three cell types (Supplementary Fig. 7A–C, and Supplementary Table 3 to 5). This finding was further confirmed by western blot analysis, which indicated direct binding between YTHDF1 and *PSEN1* mRNA in BMSCs, HEPM cells and DPSCs (Fig. 6B).

Next, the interaction between YTHDF1 and *PSEN1* mRNA was assessed via RNA immunoprecipitation (RIP) (Fig. 6C). The enrichment of *PSEN1* PCR products was observed in YTHDF1 samples compared to the control samples, validating the direct binding of YTHDF1 to *PSEN1* mRNA (Fig. 6D). As an important m^6^A reader, YTHDF1 recognizes both G(m^6^A)C and A(m^6^A)C RNAs as ligands without sequence selectivity and mediates the expression of m^6^A-modified target genes by enhancing the stability of RNA [30]. To measure the half-life of the *PSEN1* transcript upon the modulation of YTHDF1 expression, cells were treated with the transcription inhibitor actinomycin D. Consistently, YTHDF1 knockdown led to a significant decrease in the half-life of the *PSEN1* transcript, whereas YTHDF1 overexpression induced a noticeable increase in its half-life (Fig. 6E–H). These findings collectively demonstrate that METTL3-mediated m^6^A modification controls PSEN1 expression by regulating its mRNA stability in a YTHDF1-dependent manner.

### PSEN1 interacts with β-catenin and regulates Wnt/β-catenin signaling via METTL3-mediated m^6^A modification

PSEN1 is a transmembrane protein that controls the activity of several signaling pathways in subcellular compartments [31]. To assess the proteins that may interact with PSEN1, we performed co-immunoprecipitation (co-IP) coupled with MS analysis and found that five proteins could bind to PSEN1 in BMSCs, HEPM cells and DPSCs (Fig. 6I, Supplementary Fig. 7D, and Supplementary Table 6 to 11). Notably,β-catenin was the most likely protein to bind PSEN1 according to the protein-protein interaction (PPI) prediction with the STRING and GEMANIA databases (Supplementary Fig. 7E, F). Representative MS spectra of β-catenin are shown in Supplementary Fig. 7G. Subsequent co-IP analysis, supported by western blot assays, demonstrated the affinity isolated β-catenin for PSEN1 in cells (Fig. 6J). To further validate the potential cooperation between β-catenin and PSEN1 in the regulation of METTL3 expression, an immunofluorescence (IF) assay was conducted, which revealed reduced PSEN1 and β-catenin staining levels in stable METTL3 knockdown cells, as compared to control cells. Additionally, colocalization of PSEN1 and β-catenin was detected in the cytoplasm and nucleus of the above three cell lines (Fig. 6K), which indicated that β-catenin could directly bind to the PSEN1 protein (Fig. 6L).

**Fig. 7:**
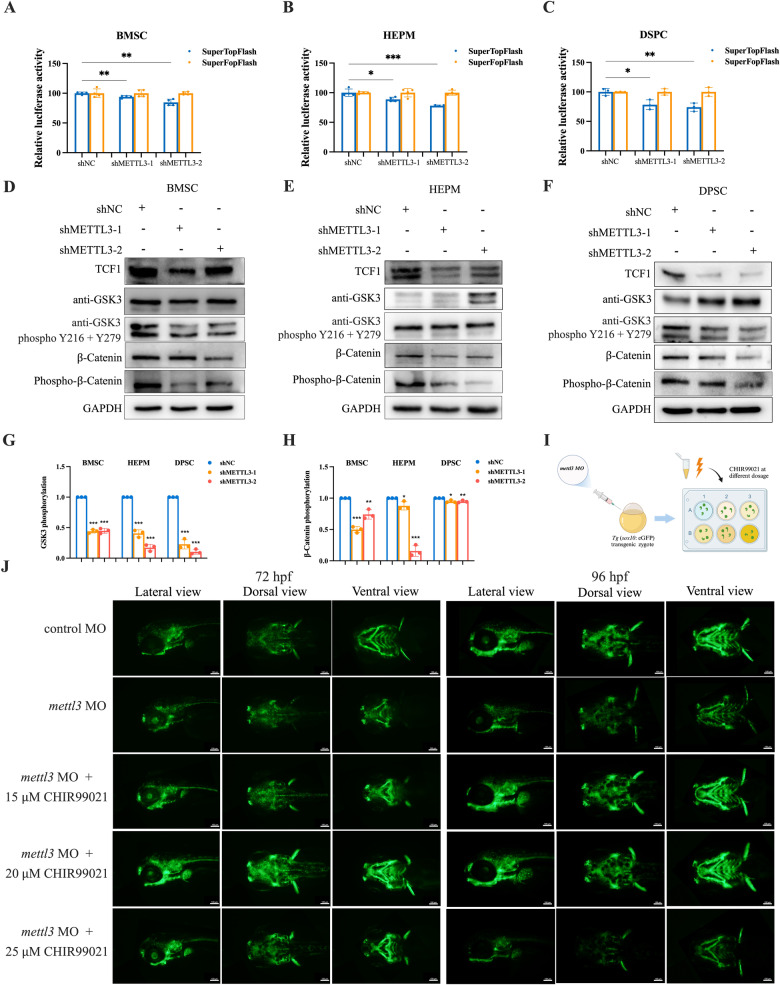
METTL3 deficiency inhibits Wnt/β-catenin signaling and Wnt/β-catenin activation partially alleviates the phenotypes of *mettl3* morphants. **A**–**C** Dual luciferase assay demonstrating the effect of SuperTop/SuperFop reporter activity in BMSCs, HEPM cells, and DPSCs transfected with the shMETTL3 vector. **D**–**F** Western blot showing the protein levels of TCF1, GSK3, phosphorylated-GSK3, β-catenin, and phosphorylated-β-catenin in control and stable METTL3 knockdown cells, GAPDH was used as a loading control. **G**, **H** Quantitative analyses of the relative expression of phosphorylated GSK3 and β-catenin. **I** Schematic of the experimental design to assess the effect of CHIR99021 at different doses. **J** Wnt/β-catenin activation partially alleviates the phenotypes of *mettl3* morphants. Results were presented as mean ± SD of three independent experiments. *P < 0.05, **P < 0.01 or ***P < 0.001 indicates a significant difference between the groups.

Given that β-catenin is critical for the Wnt/β-catenin signaling pathway, SuperTopFlash/SuperFopFlash reporters were constructed to investigate whether METTL3 modulates this pathway by regulating PSEN1. As expected, METTL3 knockdown markably decreased TOP/FOP transcriptional activity, potentially inactivating Wnt/β-catenin signaling (Fig. 7A–C). We next determined the protein levels of several key genes within this pathway. The levels of β-catenin and TCF1 were decreased after METTL3 knockdown, while METTL3 knockdown upregulated the level of GSK3 (Fig. 7D–F). Compared with that in control cells, the phosphorylation of GSK3 in METTL3-knockdown cells was significantly lower after normalization to total GSK3, but only a decrease trend was detected for phosphorylation of β-catenin in METTL3 knockdown cells normalized to the total β-catenin (Fig. 7D–H). Overall, reduced METTL3-mediated m^6^A methylation can lead to a decrease in the binding of the β-catenin to PSEN1, thus inhibiting Wnt/β-catenin signaling.

### Mitigation of disease-associated phenotypes through Wnt/β-catenin signaling pathway activation by GSK3 treatment

We then explored whether pharmacological activation of Wnt/β-catenin signaling could rescue the craniofacial malformations in the *mettl3* morphants (Fig. 7I). *Tg(sox10:eGFP)* zebrafish larvae were treated with CHIR99021, the most selective reported inhibitor of GSK3, at 15, 20 or 25 μM respectively [32, 33]. The addition of CHIR99021 partially rescued NCC development and Sox10 expression in *mettl3*-deficient embryos in a dose-dependent manner, whereas the zebrafish embryos with *mettl3* deficiency exhibited a weaker expression of Sox10 accompanied by severe developmental abnormalities at concentrations of 25 μM of CHIR99021 (Fig. 7J and Supplementary Fig. 8A, B). Moreover, in vitro data showed that 2.5, 5 or 10 μM CHIR99021 partially reversed the inhibition of cell proliferation and migration induced by METTL3-silencing, especially at a concentration of 5 μM CHIR99021 (Supplementary Fig. 9-10). These results suggest that craniofacial anomalies in *mettl3* morphants are associated with downregulated Wnt/β-catenin signaling, which can be partially alleviated by promoting Wnt/β-catenin signaling.

### Mettl3 deficiency alters motor behavior in zebrafish larvae

As PSEN1 was reported to be critical for appropriate embryonic neurogenesis [34], we further confirmed whether the m^6^A modification of *psen1* induced by Mettl3 affected the locomotor behavior of zebrafish larvae by a photoperiod stimulation test (Supplementary Fig. 11A). Records of larval motion trails showed that compared with controls, *mettl3* MO zebrafish embryos at 144 hpf exhibited hypoactive behavior. In addition, *mettl3* knockdown resulted in decreases in total swimming distance and speed in comparison to those in the control groups during dark-light transition stimulation (Supplementary Fig. 11B–E). Remarkably, we found similar activity maps after the co-injection of *mettl3* or *psen1* mRNA and treatment with 20 μM CHIR99021, which rescued the activity for voluntary movements.

## Discussion

As the most common RNA modification, m^6^A modification plays an essential role in various diseases, spanning cancers to developmental disorders [35]. The dynamic regulation of m^6^A methylation is governed by three types of regulatory factors, methyltransferases, demethylases, and reader proteins, which regulate RNA splicing, translation, export, decay, and stability. Through bioinformatic analyses and a literature search, METTL3 emerged as a potential methyltransferase closely related to craniofacial development. Operating within the methyltransferase complex in mammalian cells, METTL3 methylates its specific target transcripts and participates in various physiological processes encompassing embryonic development, brain development, and cell reprogramming [36, 37]. Cai et al. reported that METTL3 directly interacts with ACLY and SLC25A1, affecting the glycolytic pathway, and thus regulates the osteogenic differentiation of DPSCs [38]. Our present study explored the outcomes of *mettl3* knockdown, uncovering several craniofacial developmental defects, including shorter palatoquadrate and mandible lengths, decreased numbers of iridophores, aberrant tooth formation and hypoactive behavior, in zebrafish embryos. Moreover, these phenotypes were significantly rescued by *mettl3* mRNA, underscoring the critical role of METTL3 in embryonic craniofacial developmental processes.

Using MeRIP-seq and RNA-seq, the differentially expressed genes with down-regulated m^6^A levels were mainly related to somite development and the Wnt signaling pathway via enrichment analysis. The formation of somites during early embryogenesis is a fundamental and conserved feature of all vertebrate species and results in the metameric organization of the vertebrae and the associated skeletal muscles, nerves, and blood vessels [39]. Our in vivo experiments identified *PSEN1* within the Wnt signaling pathway as a pivotal target of METTL3. Recent studies have characterized PSEN1 as a transmembrane protein with nine transmembrane domains connected to hydrophilic loops in either the extracellular space or the cytosol [31, 40]. Cerebral organoids derived from Alzheimer’s disease induced pluripotent stem cell lines with mutant *PSEN1* showed altered development and premature differentiation during human stem cell neurogenesis [41, 42]. However, the landscape of post-transcriptional *PSEN1* mRNA regulation in craniofacial development has not been determined. In this study, we revealed the METTL3-*PSEN1* mRNA interaction, underscoring the crucial role of m^6^A modification in regulating the PSEN1 level. Moreover, *PSEN1* overexpression partially reversed the inhibition of proliferation and migration in METTL3-silenced cells. Co-injection of *mettl3* or *psen1* mRNA rescued *mettl3* knockdown-induced hypoactive behavior in larvae. These findings suggest a previously uncharacterized mechanism of PSEN1 in the regulation of m^6^A modification.

Recent studies have indicated how m^6^A modification affects transcript translation, stability and intercellular transcriptome switching—a pivotal mechanism in steering proper development [22]. Neural crest formation commences during early vertebrate development, culminating in migration that contributes to a wide variety of derivatives including craniofacial cartilage and bone, neurons of the peripheral nervous system, and melanocytes [43, 44]. As master transcription factor of the neural crest, Sox10 is necessary for the proliferation, migration, and differentiation of multipotent neural crest during embryogenesis [45, 46]. Consistent with the expression of zebrafish *sox10* in early cranial NCCs from 10 to 16 hpf [47], both *mettl3* and *psen1* exhibited similar expression patterns based on the spatial transcriptomics data obtained during zebrafish embryogenesis and early gene expression detection. Notably, the rescue of Sox10 expression alongside abnormal craniofacial phenotypes induced by *mettl3* or *psen1* mRNA indicates that *mettl3*-mediated m^6^A modification regulates Sox10, possibly through its effect on *psen1*, thereby influencing craniofacial developmental events in vertebrate embryogenesis.

The well-established role of m^6^A modification in the regulation of mRNA decay, translation efficiency, mRNA splicing, and export by binding to the reader protein has been extensively described [48]. RNA pull-down and RIP assays demonstrated that YTHDF1, but not the other readers, could bind to *PSEN1* mRNA. YTHDF1 is an important m^6^A modification reader, and its primary function is to promote protein translation or regulate the stability of m^6^A-modified mRNAs [49, 50]. Notably, YTHDF1 knockdown attenuated ameliorated pulmonary artery smooth muscle cell proliferation, phenotypic switching, and the development of pulmonary hypertension by enhancing MAGED1 translation both in vivo and in vitro [51]. Another study found that YTHDF1 enhanced the stability of c-Myc mRNA catalyzed in an m^6^A-dependent manner, thereby promoting c-Myc expression [24]. Our results are consistent with this mechanism, as we found that the m^6^A modification of *PSEN1* enhanced mRNA stability in a YTHDF1-dependent manner. The current study substantiates the ability of METTL3 to regulate the m^6^A modification of *PSEN1*, while YTHDF1 enhances the mRNA stability of m^6^A-modified *PSEN1*.

The crucial role of β-catenin within Wnt1 expressing cell lineages has been well established, as its inactivation has been correlated with severe craniofacial defects [52, 53]. The potential involvement of PSEN1 in Wnt signaling through the regulation of β-catenin stability positions it as a candidate for in-depth mechanistic exploration [40]. In light of its central role, β-catenin serves as an intracellular messenger for canonical Wnt signaling, a pathway instrumental in activating and maintaining the factors necessary for NCC development [54, 55]. Similarly, the significant reduction of β-catenin in stable METTL3 knockdown cells impeded Wnt pathway activation, concurrently suppressing Sox10 expression via METTL3-YTHDF1-dependent silencing of *PSEN1* mRNA. Importantly, pharmacological activation of the Wnt/β-catenin pathway partially alleviated the phenotypes of *mettl3* morphants, rescued the activity of voluntary movements and reversed the decreases in cell proliferation and migration induced by METTL3 silencing, thereby reinforcing the role of Wnt signaling in this process.

In summary, we identified and characterized *PSEN1* as a novel m^6^A-regulated target in craniofacial development. Mechanistically, METTL3-mediated m^6^A modification controls the expression of PSEN1 by regulating its mRNA stability in a YTHDF1-dependent manner. PSEN1 regulates Wnt/β-catenin signaling by binding to β-catenin, thereby influencing craniofacial developmental events via METTL3-mediated m^6^A modification. Our study highlights the functional importance of the METTL3/YTHDF1/PSEN1/β-catenin signaling axis, which provides new insights into the exploration of the underlying regulation mechanism for craniofacial development.

## Materials and methods

### Zebrafish maintenance

Zebrafish (Tubingen) and *Tg(sox10:eGFP)* transgenic zebrafish were maintained at 28 °C using standard protocols. Zebrafish embryos were cultured in E3 medium supplemented with 0.01 mg/L methylene blue and collected at the 1-cell stage. All the experimental protocols were approved by the Animal Ethics Committee of the Affiliated Stomatology Hospital of Nanjing Medical University. For all zebrafish experiments, embryos were collected and sorted into treatments blindly to ensure appropriate randomization.

### MO-mediated knockdown, rescue, and treatment with the GSK3 inhibitor CHIR99021

MO antisense oligonucleotides targeting *mettl3* (forward: 5’-ACCCAGAGCTAGAGAAGAGG-3’, reverse: 5’-CACAGAACTCCTGAACTTGA-3’, RC: TCAAGTTCAGGAGTTCTGTG) were synthesized by GeneTools to block the translation of *mettl3* mRNA. A 5-base-pair mismatch MO served as a control. The *mettl3* MO (8 ng) or control MO (8 ng) was microinjected into zebrafish embryos at 1-cell stage as previously described [56]. For rescue experiments, zebrafish *mettl3* or *psen1* mRNA vectors (100 ng/μl) were co-injected with *mettl3* MO at 1-cell stage. Knockdown and rescue of *mettl3* were further confirmed by qRT-PCR. *Tg(sox10:eGFP)* zebrafish larvae injected with the *mettl3* MO were exposed to GSK3 inhibitor CHIR99021 (15, 20 or 25 μM) during zebrafish embryonic development [32, 33]. Embryos were imaged using a Nikon SMZ800N stereomicroscope (NIKON Corporation, Tokyo, Japan).

### Alcian blue and alizarin red staining

To assess morphological craniofacial changes, zebrafish embryos at 144 h post-fertilization (hpf) were randomly collected and fixed in 95% ethanol overnight. Subsequent staining involved incubating the embryos with 0.02% alcian blue (A8140, Solarbio, Beijing, China) and 0.5% alizarin red overnight, followed by soaking in distilled water for 10 min and bleaching with 1.5% H_2_O_2_/1.5% KOH for 3 h until soft tissue transparency was achieved. The embryos were washed twice in 50% glycerol at room temperature. Phenotypic assessments included quantitative analysis of mouth opening distance, palatoquadrate and mandible lengths, as well as ethmoid plate and mandible cartilage widths.

### RNA extraction and quantitative real-time PCR (qRT-PCR)

Total RNA was isolated from zebrafish embryos and cells using an RNA Extraction Kit following the manufacturer’s instructions (RX112, Vazyme, Nanjing, China). cDNA templates were synthesized from RNA using the PrimeScript™ RT Regent Kit (RR036A, Takara Bio, Shiga, Japan). qRT-PCR analysis was performed with a SYBR Green RT-PCR Kit (Q712-02, Vazyme, Nanjing, China) and a Roche Light Cycler 480 II system (Roche, Switzerland). The sequences of primers used are listed in Supplementary Table 12. *GAPDH* or *gapdh* was used as the internal control in all the experiments.

### m^6^A dot blot assay

Total RNA from zebrafish embryos or cells was denatured at 95 °C for 5 min and spotted onto nylon membranes (GE Healthcare, USA). After ultraviolet crosslinking, the membranes were incubated with an m^6^A antibody (1:250, CST, #56593) overnight. Following incubation with horseradish peroxidase-conjugated anti-rabbit IgG secondary antibody for 1 h at room temperature and enhanced chemiluminescence development, the membranes were dyed with 0.02% methylene blue (M196499, Aladdin, Shanghai, China) and 1× TAE (ST716, Beyotime, China) and scanned to determine the input RNA content.

### Western blot assay

Total protein was extracted from zebrafish embryos and cultured cells with RIPA lysis buffer supplemented with 0.5% PMSF and protease inhibitors. Equal amounts of protein were separated on 10% sodium dodecyl sulfate–polyacrylamide gel electrophoresis (SDS‐PAGE) and transferred to polyvinylidene fluoride (PVDF) membranes (Millipore, Darmstadt, Germany). The membranes were blocked with 5% non-fat milk for 2 h at room temperature and then immunostained with primary antibodies overnight at 4 °C. The primary antibodies used in this study were as follows: anti-Sox2 (1:1000, GeneTeX, #GTX627404), anti-Sox3 (1:1000, GeneTeX, #GTX132494), anti-Sox10 (1:1000, GeneTeX, #GTX128374), anti-β-actin(1:1000, GeneTeX, #GTX629630), anti-METTL3 (1:1000, Abcam, ab195352), anti-PSEN1 (1:1000, CST, #5643), anti-YTHDF1 (1:1000, Proteintech, 17479-1-AP) and anti-GAPDH (1:1000, Beyotime, AG019), anti-TCF1 (1:1000, CST, #2203 T), anti-phospho-β-catenin (1:1000, CST, #9561), anti-GSK3(1:5000, Abcam, ab40870), and anti-GSK3 phospho Y216 + Y279 (1:1000, Abcam, ab68476). Subsequently, the membranes were incubated with the corresponding secondary antibodies for 1 h, and washed with TBST three times, followed by detection with the ECL chemiluminescent detection system (P10100, NcmECL Ultra, Suzhou, China).

### Data sources

Processed data from spatiotemporal transcriptomic atlases of mouse organogenesis were obtained from MOSTA (https://db.cngb.org/stomics/mosta) [57]. Dynamic spatiotemporal transcriptomic atlas during zebrafish embryogenesis was downloaded from the ZESTA database (https://db.cngb.org/stomics/zesta/) [58]. The single-cell dataset from bone marrow in the Tabula Sapiens database was available from the Gene Expression Omnibus (accession numbers GSE201333).

### Study cohort, genotyping, quality control, and phenotyping

The study cohort comprised a skeletal sagittal malocclusion cohort that underwent genotyping using the Illumina Global Screen Array (GSA) by Genergy Biotechnology Co. Ltd, that included 471 cases (0.7$^\circ \le$ ANB $\le$ 4.7°) and 664 healthy controls (ANB < 0.7° or ANB > 4.7°) [59, 60]. Additionally, FinnGen summary statistics, encompassing tooth development and eruption data, and information on hard palate cleft, were imported from a researcher-accessible source (https://r9.finngen.fi) in July 2023. Variants and samples were excluded under the following conditions: (1) call rates less than 95%. (2) minor allele frequency (MAF) lower than 0.01 and (3) genotype distribution deviating from Hardy-Weinberg equilibrium (*P* < 1.00 × 10^−5^). All the study protocols were approved by the Affiliated Stomatology Hospital of Nanjing Medical University (PJ2020-079-001).

### Cell culture, lentiviral transduction, expression plasmids, short interfering RNAs, cell transfection and treatment

Human bone marrow mesenchymal stem cells (BMSCs) were purchased from Cyagen Biosciences (Guangzhou, China). Adherent BMSCs were cultured in BMSC growth medium (Cyagen Biosciences, Inc., Guangzhou, China) at 37 °C with 5% CO_2_ and were passaged after reaching 80% confluence. Human embryonic palatal mesenchymal (HEPM) cells were cultured in alpha Dulbecco’s modified Eagle’s medium (α-MEM) containing 10% fetal bovine serum (Gibco, USA), 100 μg/ml streptomycin (Gibco, USA), and 100 U/ml penicillin (Gibco, USA) at 37 °C in 5% CO_2_. Similarly, human dental pulp stromal cells (DPSCs) were cultured in α-MEM containing 10% fetal bovine serum, 100 μg/ml streptomycin, and 100 U/ml penicillin at 37 °C in 5% CO_2_. Cells from passages 2-7 were used in subsequent experiments.

For stable knockdown, shRNA oligos targeting *METTL3* were designed (GenePharma, Shanghai, China). The lentivirus containing LV3-GFP was purchased and packaged according to the manufacturer’s instructions. The indicated cell lines were transduced with the shMETTL3-1/shMETTL3-2 or empty vector lentiviruses containing medium for 48 h, followed by puromycin selection.

Small interfering RNA (siRNA) duplexes targeting human *YTHDF1* were synthesized by GenePharma (China). Human *PSEN1* (NM_000021.4) and *YTHDF1* (NM_017798.4) was cloned through PCR and constructed in the pEX-3 vectors for overexpression respectively. The designated siRNAs and plasmids were transfected into cells with Lipofectamine 2000 (Invitrogen, USA) according to the manufacturer’s protocols. Thereafter, the transfection efficacy was tested by qRT-PCR and western blotting. The sequences of the siRNAs, vectors, and shRNAs used are listed in Supplementary Table 12.

To explore the impact of pharmacological activation of Wnt/β-catenin signaling on cell phenotypes, the cells were treated with the GSK3 inhibitor CHIR99021 (2.5, 5 or 10 μM) based on previous studies [61, 62].

### Cell proliferation and migration assays

To ascertain cell proliferation, approximately 3000 transfected cells were seeded in 96-well plates as monolayers. Cell viability was measured at various time points using the Cell Counting Kit-8 (CCK8, Kumamoto, Japan) on a microplate reader (SpectraMax 190, Molecular Devices, USA) at 450 nm (OD450). Moreover, EdU reagent was used for proliferation assessment following the manufacturer’s instructions. Fluorescence microscope (Leica, DM4000) was utilized for sample analysis. Three replicates were performed for each sample.

Additionally, transwell assays were performed to evaluate cell migration. The cell suspension was seeded in the upper layer of the transwell chambers (8 μm pore size, Millipore, Darmstadt, Germany) in serum-free α-MEM. The lower chambers contained 600 μl of complete α-MEM containing 20% FBS. After 24-36 h of incubation, cells that migrated through the membrane were fixed with 4% paraformaldehyde, stained with crystal violet, and counted in four randomly chosen fields under a microscope (Nikon, SMZ800N, Tokyo, Japan).

### Public sequencing datasets and RNA-seq

The MeRIP-seq and RNA-seq datasets of mettl3 morphants, accessible through the Gene Expression Omnibus (GEO) public database under accession number GSE89655, were analyzed using methodologies consistent with those used in a previous study [26]. To ensure data quality, we employed FastQC and RseQC to verify the sequence quality. Subsequently, the HISAT2 tool was utilized for the alignment of reads to the reference genome from *Danio rerio* (GRCz10). Peak calling and differential peak analysis were performed with the R package exomePeak2, and peak annotation was performed through intersection with gene architecture using the R package ANNOVAR. For the RNA-seq analysis of mettl3 morphants, after undergoing quality control, the clean reads were mapped to each Ensembl gene (GRCz10). Raw reads were generated via illumina Hiseq. Differential gene expression was assessed using the DESeq2 package in R software.

For the RNA-seq assay, total RNA was extracted from control or METTL3-knockdown BMSCs and then subjected to library construction and sequencing. After quality control, clean reads were mapped to human reference genome (hg38) and raw counts were generated via Illumina NovaSeq. Differentially expressed genes (DEGs) were selected based on the following criteria: |log_2_(fold change) | >1.3 and *P* value < 0.05. The volcano map of DEGs was generated with the R package ggplot2 v3.4.2.

### Functional enrichment analysis

Enrichment analysis of DEGs with downregulated m^6^A levels was analyzed based on Metascape online tool (http://metascape.org/gp/index.html#/main/step1). For the identification of perturbed pathways, KEGG pathway analysis was performed by employing the clusterProfiler package, with a significance threshold of *P* < 0.05 based on marker genes.

### MeRIP-qPCR

To quantify m^6^A-modified specific gene levels, total RNA was extracted from cells. A portion of the RNA sample was reserved as the input control. The remaining RNA was subjected to an overnight incubation at 4 °C with anti-m^6^A-antibody-conjugated or IgG-conjugated beads in immunoprecipitation buffer supplemented with RNase inhibitor. The m^6^A-containing RNA samples were immunoprecipitated and eluted from the beads. Subsequently, both the input and IgG control samples, alongside the m^6^A-immunoprecipitated samples were isolated and purified for qPCR analysis, facilitating the detection of the target mRNAs.

### DAA treatment

To evaluate the functional impact of *METTL3*-mediated m^6^A modification on the regulation of *PSEN1*, we employed the methylation inhibitor DAA [63]. Cells were cultured in 6-well plates and treated with various concentrations of DAA (600 and 700 μmol). Cells were harvested 48 h after DAA treatment and total RNA was extracted for qRT-PCR assays.

### Motif analysis

We employed SRAMP (http://www.cuilab.cn/sramp), a powerful tool for predicting m^6^A modifications at different loci with varying confidence thresholds. This allowed us to predict the motif of m^6^A modifications [64].

### Luciferase reporter assay

To evaluate the impact of m^6^A sites on *PSEN1* expression, cDNA containing the 3’UTR sequence of *PSEN1* was inserted into a luciferase reporter vector (pcDNA3.1 vector) as the wild-type construct. Predicted m^6^A sites (sequence: GGACC) in *PSEN1* were replaced with random sequence (TTGAG) to generate the mutant plasmids. *METTL3*-knockdown and control cells were transfected with wild-type or mutated *PSEN1* reporter plasmids. After 48 h of transfection, we measured the relative luciferase activity using the Duo-Lite Luciferase Assay System (DD1205-01, Vazyme, Nanjing, China). All the experiments were performed in triplicate.

### SELECT assay for the single-base detection of m^6^A modification

To detect the m^6^A modification at the single-site level, we applied the single-base elongation- and ligation-based qPCR amplification method (SELECT) [65]. The SELECT assays were performed with an Epi-SELECT™ m^6^A Site Identification Kit (Epibiotek, Guangzhou, China) [66]. In total, the probe pairs targeting the sites at the *PSEN1* transcript and primers used for the SELECT assays are listed in Supplementary Table 12. The qPCR reaction was performed using ChamQ Universal SYBR qPCR Master Mix (Vazyme, Q711) with the Roche Lightcycler 480 Instrument II system.

### RNA pull-down and mass spectrometry (MS) analysis

Specific probes for *PSEN1* and corresponding antisense probes were synthesized by GenePharma (China). Total protein was extracted from the cells. The RNA pull-down assay was performed according to the manufacturer’s instructions using the Pierce Magnetic RNA–Protein Pull-Down Kit (Thermo Fisher Scientific). Co-precipitated proteins were eluted from the beads using loading buffer. The recruited proteins were visualized by silver staining using the Fast Silver Stain Kit and further characterized through MS analysis. The expression of the identified proteins was confirmed by western blotting.

### RIP assays

We conducted RIP assays using the Magna RIP kit (17-700, Millipore Magna, USA) according to the manufacturer’s guidelines. Approximately 1 × 10^7^ cells were collected and lysed with lysis buffer. The supernatant was incubated with magnetic beads coupled with either anti-YTHDF1 antibody (Proteintech, 17479-1-AP) or control IgG at 4 °C overnight. The bead-RNA-protein complex was washed and treated with Proteinase K. The precipitated RNA was further purified and quantified by qRT-PCR.

### RNA stability assay

YTHDF1-knockdown, YTHDF1-overexpression, and control cells were exposed to 5 μg/mL actinomycin D (M4881, Abmole Bioscience, USA) to suppress mRNA transcription. Cells were collected at various time points (0, 2, 4, 6, 8, and 10 h posttermination) and total RNA was extracted. The remaining *PSEN1* mRNA level was quantified using qRT-PCR. Half‐life (t_1/2_) of *PSEN1* mRNA was calculated according to previous methods [17, 67] and *GAPDH* was used for normalization.

### Co-IP

Cells were harvested and resuspended with ice-cold IP lysis buffer (Thermo Fisher Scientific, 88804). The lysates were centrifuged at 14,000 rpm for 10 min to obtain the supernatant containing total proteins. Then the cell lysate was mixed with PSEN1 or CTNNB1 antibody. After gently rotating at 4 °C overnight, 5 μl protein A/G agarose beads were added to each tube and incubated at 4 °C for 1–3 h. Finally, the beads were rinsed with wash buffer and heated at 95 °C for 10 min. After silver staining using the Fast Silver Stain Kit, MS analysis was carried out to identify substrate proteins that bind to PSEN1 based on their scores and masses. Western blot assay was performed to validate the interactions between bound proteins and PSEN1.

### IF staining

The METTL3-knockdown and control cells were seeded onto cover slides and incubated under standard culture conditions for 24–36 h. After an overnight culture, cells were fixed in 4% paraformaldehyde for 15 min at room temperature and then permeabilized with 0.1% Triton X-100. Subsequently, cells were incubated with appropriate primary antibodies (anti-PSEN1 1:50, CST, #5643 and anti-β-catenin 1:50, Santa Cruz, sc-7963) at 4 °C overnight and subjected to secondary antibodies conjugated with Alexa Fluor (Invitrogen) for 1 h at room temperature. DAPI containing antifade medium was used for nuclear staining. Images were captured by Nikon Imaging Software (NIS)-Elements and evaluated using ImageJ software.

### SuperTopFlash reporter assay

The SuperTopFlash/SuperFopFlash reporter assay was used to measure β-catenin–driven TCF/LEF transcriptional activation. The cells were co-transfected with the SuperTopFlash/SuperFopFlash construct (Beyotime, China), and either the METTL3 knockdown vector or the control vector using Lipofectamine 2000 transfection reagent (Life Technologies). After 48 h post-transfection, cells were lysed and luciferase activity was evaluated using a dual luciferase reporter system. Firefly luciferase activity normalized to Renilla luciferase activity was expressed as the relative fold change.

### Behavioral assays

After incubating for 144 h, the zebrafish larvae in each treatment group were transferred to the 24-well plates and the behaviors including trajectories, swimming distance, and swimming speed were assessed via a DanioVision observation chamber (Noldus Information Technology, Netherlands). Zebrafish larvae were acclimated to plate conditions for 30 min before the behavioral analysis. The 20 min light-induced visual motor response recording consisted of two alternate cycles of 5 min in the dark followed by 5 min in light. The temperature of the well plates (28 ± 0.5 °C) during the experiment was maintained by the DanioVision temperature control unit. All the video recordings were analyzed using the EthoVision XT software (Noldus Information Technology, Netherlands).

### Statistical analysis

All numerical data are presented as means ± standard deviations (SD). Pilot experiments and previously published results were used to estimate the proper sample size. Experiments were independently repeated at least three times. Differences between two groups or multiple groups were evaluated by Student’s *t*‐test, Mann-Whitney U-test and ANOVA test. Pearson’s correlation analysis was utilized to describe correlations between quantitative variables without a normal distribution. The ORs and 95% CIs for genetic associations were obtained using multivariate logistic regression analysis. Statistical analyses were calculated with R software (version 4.0.5) and the figures were plotted using GraphPad Prism 7.0 (GraphPad Software, La Jolla, CA, USA). All statistical tests were two‐sided. A two‐sided *P*-value of less than 0.05 was considered to indicate statistical significance (*P-value < 0.05, **P-value < 0.01, ***P-value < 0.001; ns, not significant).

### Supplementary information

Supplementary Figures

Supplementary Table 1

Supplementary Table 2

Supplementary Table 3

Supplementary Table 4

Supplementary Table 5

Supplementary Table 6

Supplementary Table 7

Supplementary Table 8

Supplementary Table 9

Supplementary Table 10

Supplementary Table 11

Supplementary Table 12

checklist

## Data Availability

All data needed to evaluate the conclusions in the paper are present in the paper and/or the Supplementary Materials. Accession numbers of published data used in this study are GSE201333 and GSE89655.
