# Supplementary material for: METTL3-dependent m6A modification of PSEN1 mRNA regulates craniofacial development through the Wnt/β-catenin signaling pathway
Source: Cell Death Dis. 2024 Mar 20;15(3):229. doi: 10.1038/s41419-024-06606-9 (PMC10954657; doi:10.1038/s41419-024-06606-9)
Supplement: Supplementary file 1 — Supplementary Figures [file 41419_2024_6606_MOESM1_ESM.pdf]

METTL3-dependent m6A modification of *PSEN1* mRNA regulates craniofacial development through the Wnt/β-catenin signaling pathway

Lan Ma *et al.*

*Corresponding author. Email: panyongchu@njmu.edu.cn

This PDF file includes:

Supplementary Fig. 1 to 11

Other Supplementary Material for this manuscript includes the following:

Supplementary Table 1 to 12

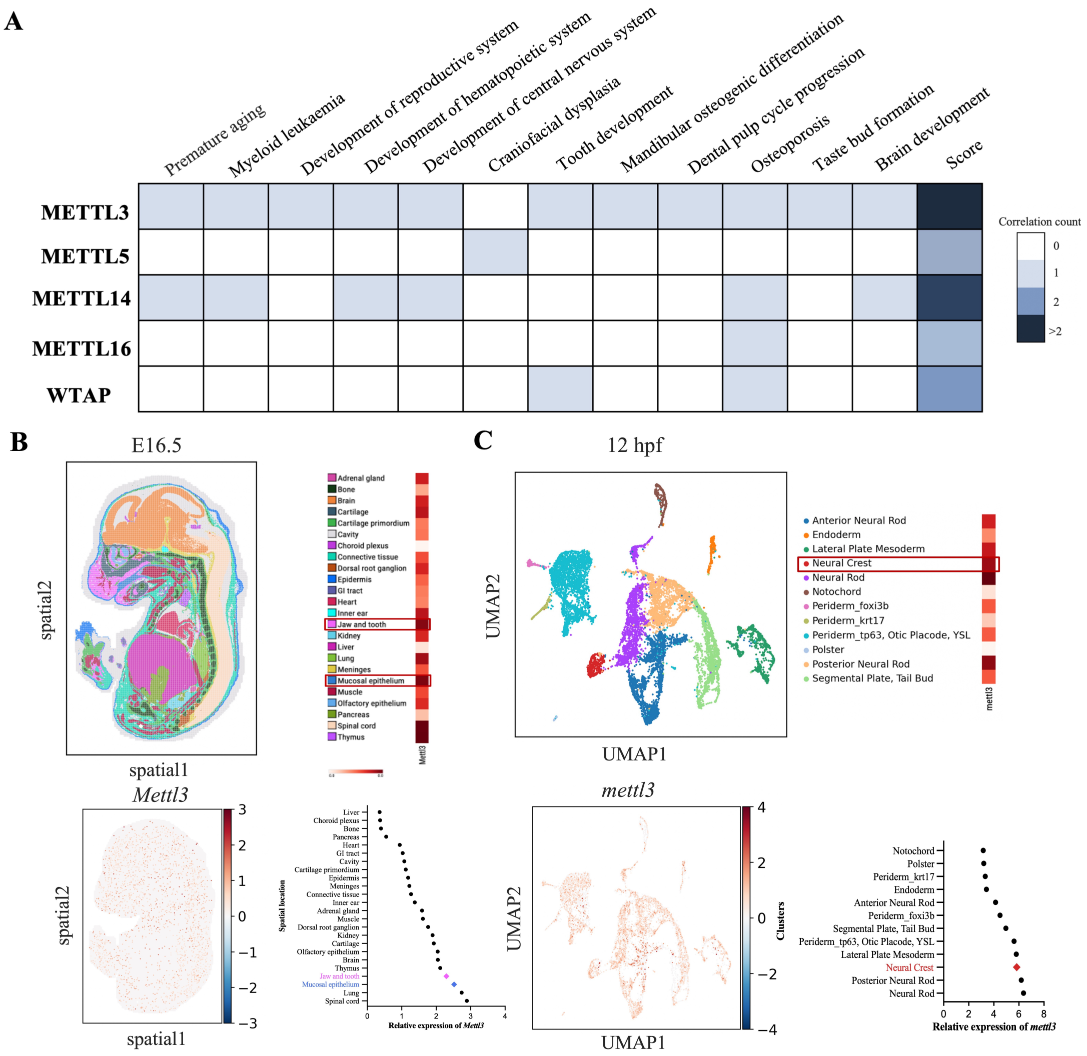

Supplementary Fig. 1

(**A**) Literature scores for m^{6}A modification based on PubMed, Google Scholar and Web of Science. (**B**) The expression of *Mettl3* in spatial transcriptome data of C57BL/6 mouse embryos at E16.5. (**C**) The expression of *mettl3* in zebrafish embryos at 12 hours postfertilization (hpf) obtained from scRNA-seq data.

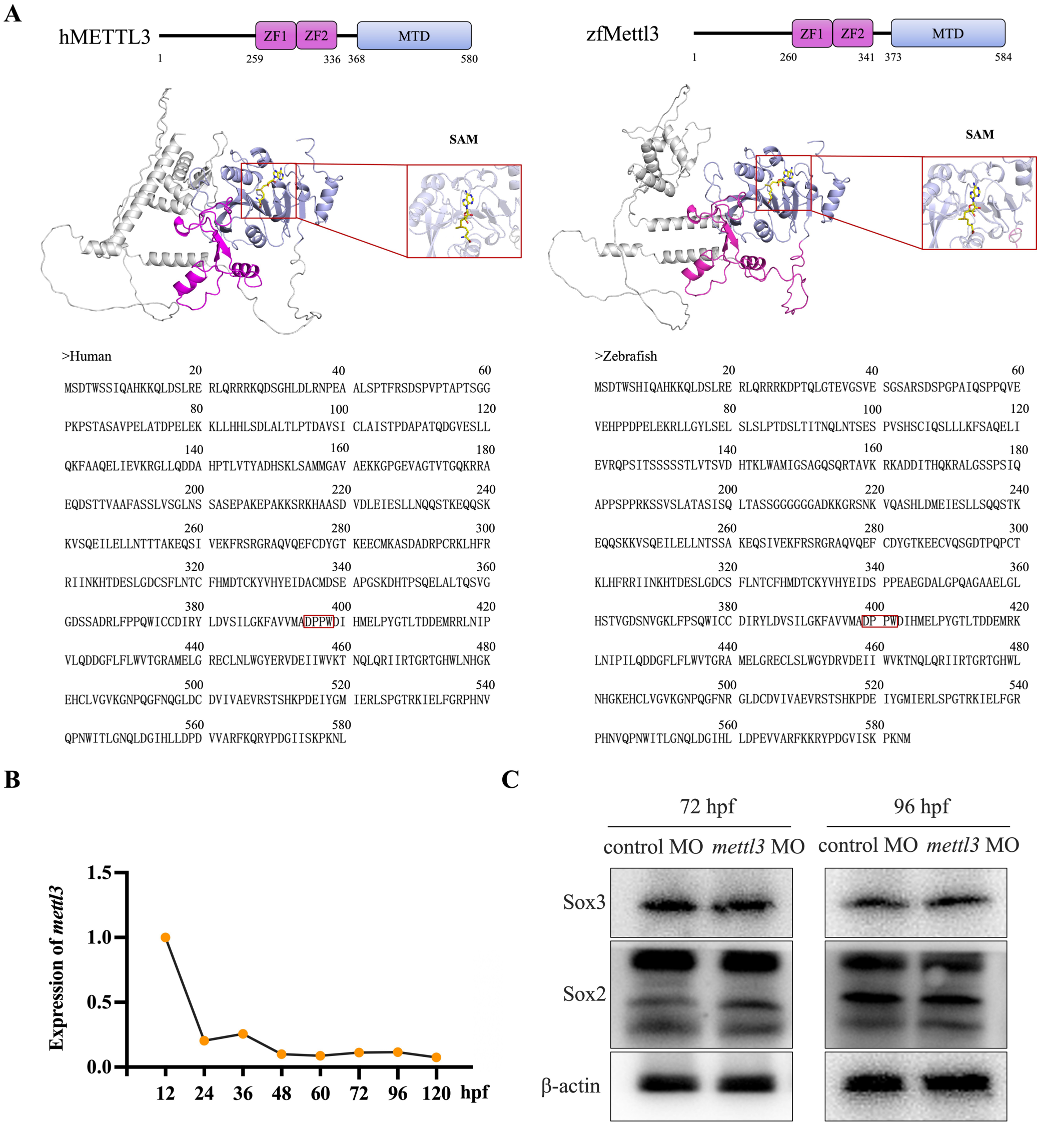

Supplementary Fig. 2

(**A**) Schematic outline and prediction of three-dimensional structure of the human METTL3 and zebrafish Mettl3 protein. The red box is a partially enlarged diagram of the S-adenosylmethionine (SAM) of METTL3. (**B**) The expression of *mettl3* in zebrafish embryos from 12 to 120 hpf. (**C**) Western blot analysis of the expression of Sox2 and Sox3 in the *mettl3*-knockdown zebrafish embryos at 72 and 96 hpf.

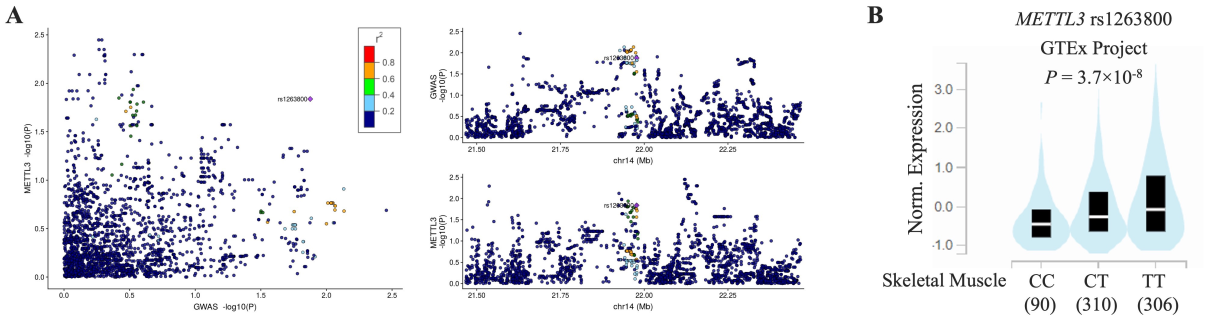

Supplementary Fig. 3

(**A**) Colocalization between GWAS signal at rs1263800 and expression QTL (eQTL) mapping using skeletal muscle. (**B**) The rs1263800 variant was significantly associated with expression of *METTL3* in skeletal muscle.

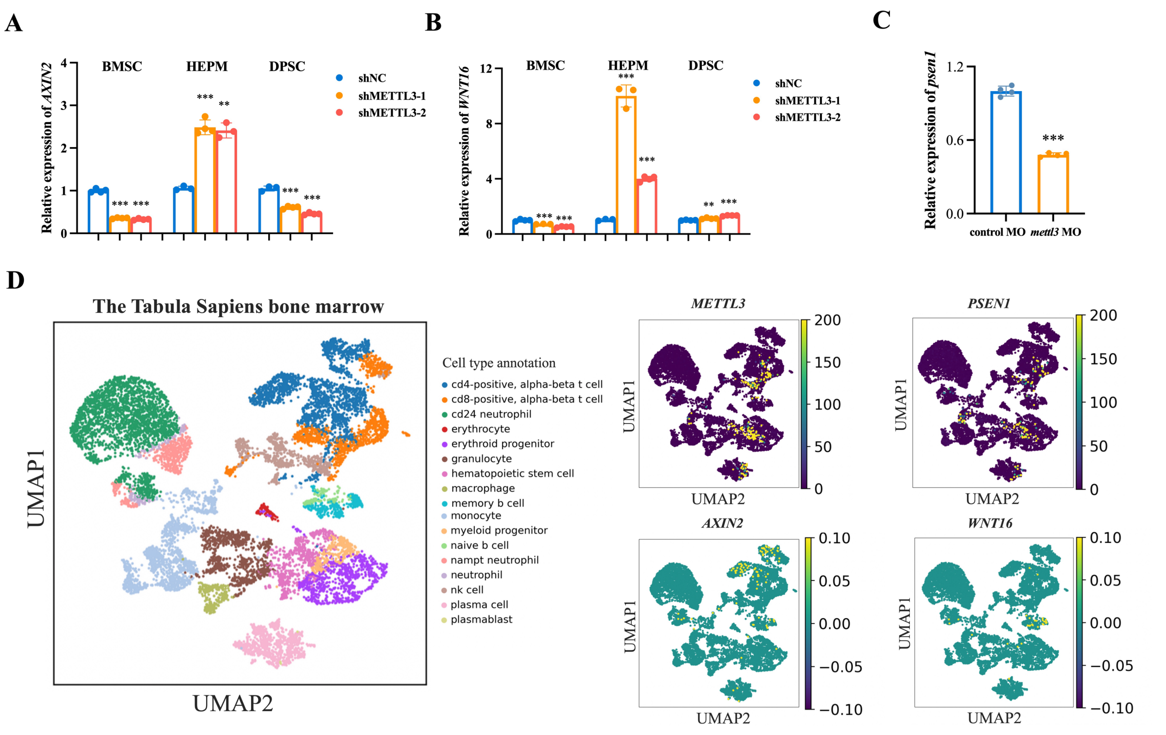

Supplementary Fig. 4

(**A** and **B**) The expression level of *AXIN2* and *WNT16* in BMSCs, HEPM cells and DPSCs with METTL3-knockdown and control groups. (**C**) The expression of *psen1* in the *mettl3*-knockdown zebrafish embryos at 72 hpf. (**D**) The expression of *METTL3*, *PSEN1*, *AXIN2* and *WNT16* during the scRNA-seq data of bone marrow in the Tabula Sapiens database. *$P < 0.05$, **$P < 0.01$ or ***$P < 0.001$ indicates a significant difference between the groups.

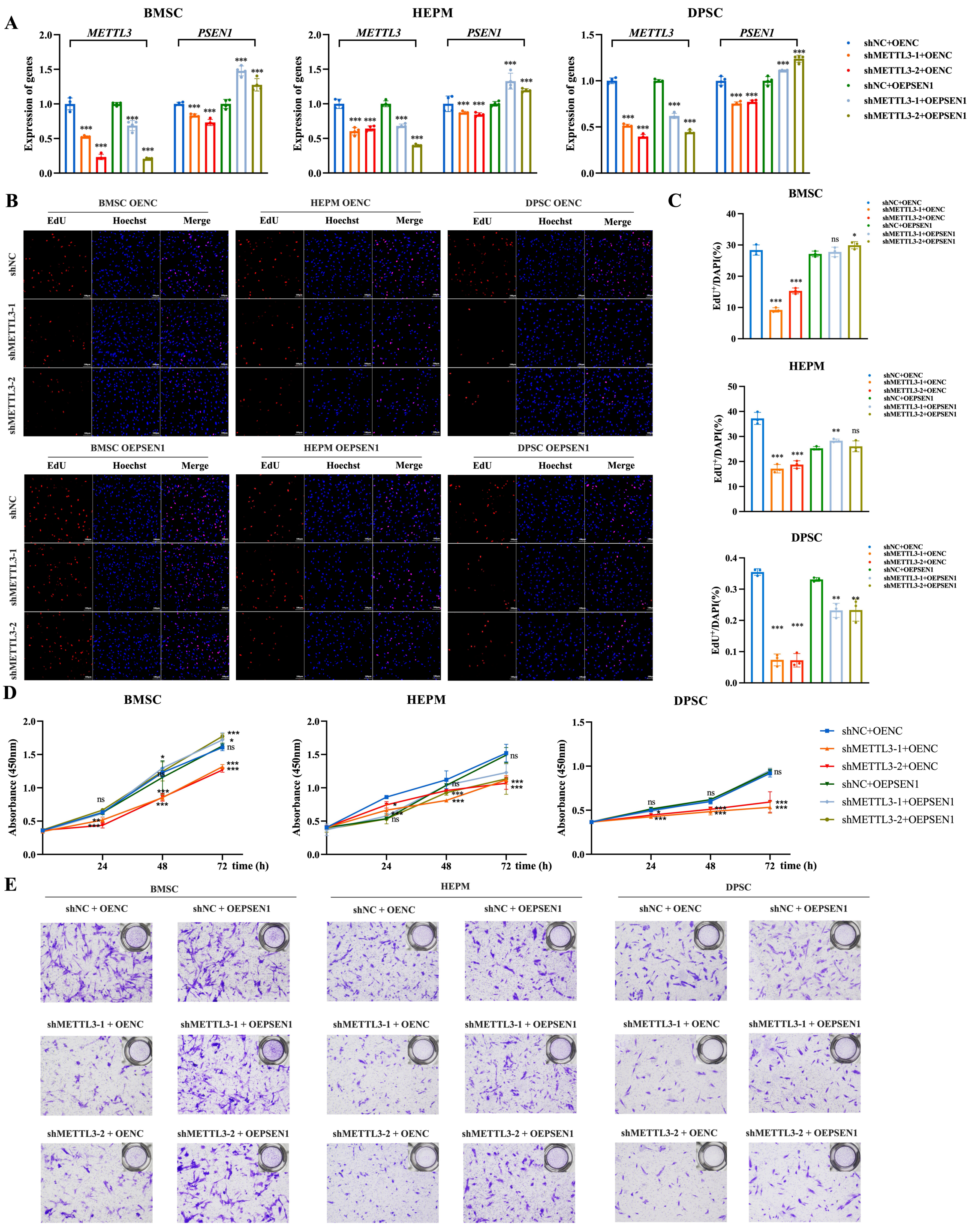

Supplementary Fig. 5

(**A**) The efficiency of *PSEN1* overexpression in BMSCs, HEPM cells and DPSCs with METTL3-knockdown and control groups. (**B** to **D**) The effect of *PSEN1* overexpression on the cell proliferation in METTL3-silenced cells measured by EdU and CCK8 assays. (**E**) The effect of *PSEN1* overexpression on the migration abilities of METTL3-silenced cells determined by a transwell assay.

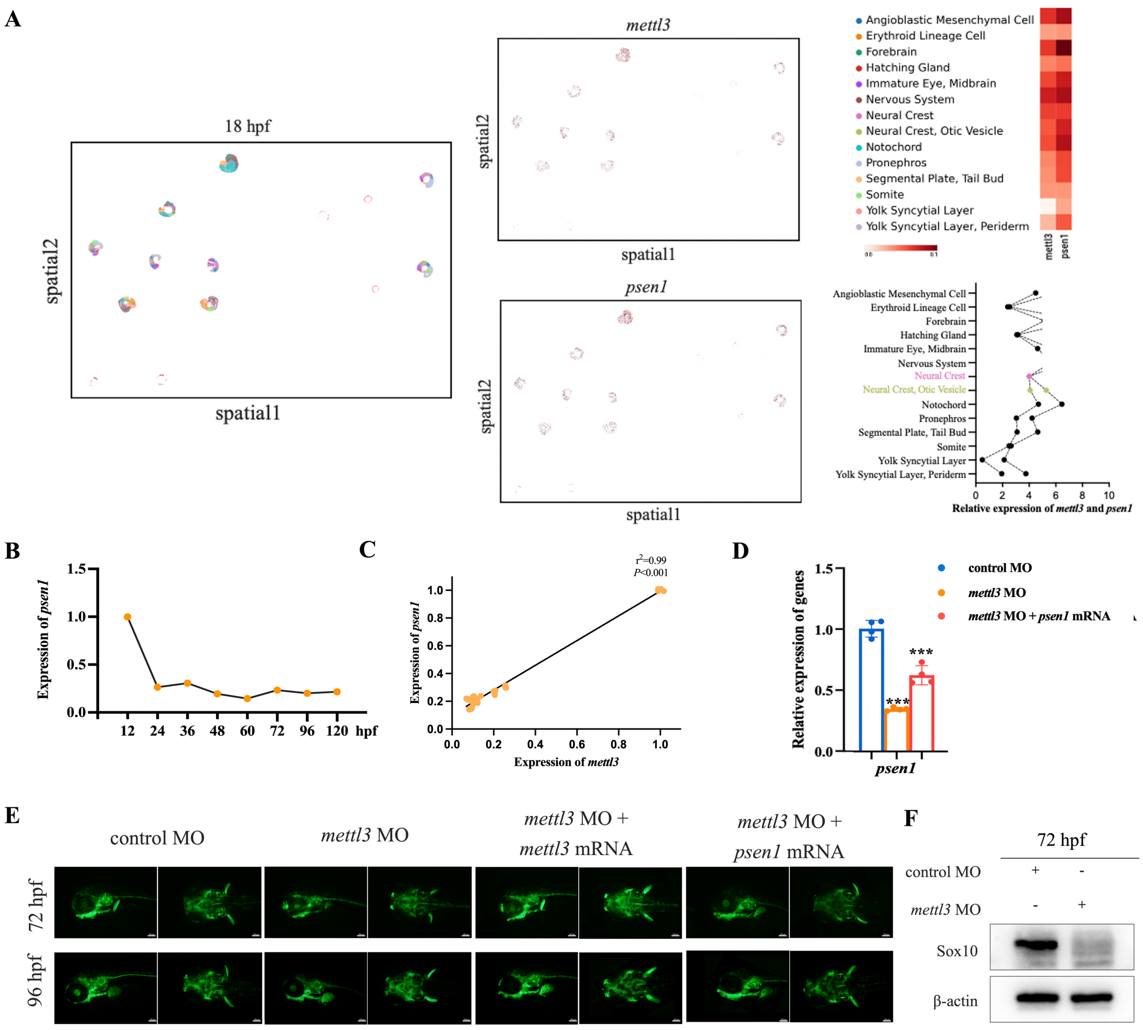

Supplementary Fig. 6

(**A**) The expression of *mettl3* and *psen1* in zebrafish embryos at 18 hpf of spatial transcriptome data. (**B**) The expression of *psen1* in zebrafish embryos from 12 to 120 hpf. (**C**) *psen1* was co-expressed with *mettl3* in zebrafish embryos from 12 to 120 hpf. (**D**) The expression of *psen1* detected in zebrafish embryos injected with control MO, *mettl3* MO, or co-injected with *mettl3* MO and *psen1* mRNA. (**E**) The effects of *mettl3* and *psen1* on *Tg*(*sox10: eGFP*) transgenic zebrafish embryos with *mettl3*-knockdown at 72 and 96 hpf from the ventral and lateral views. (**F**) Western blot analysis of the Sox10 levels in the *mettl3*-knockdown and control zebrafish embryos at 72 hpf.

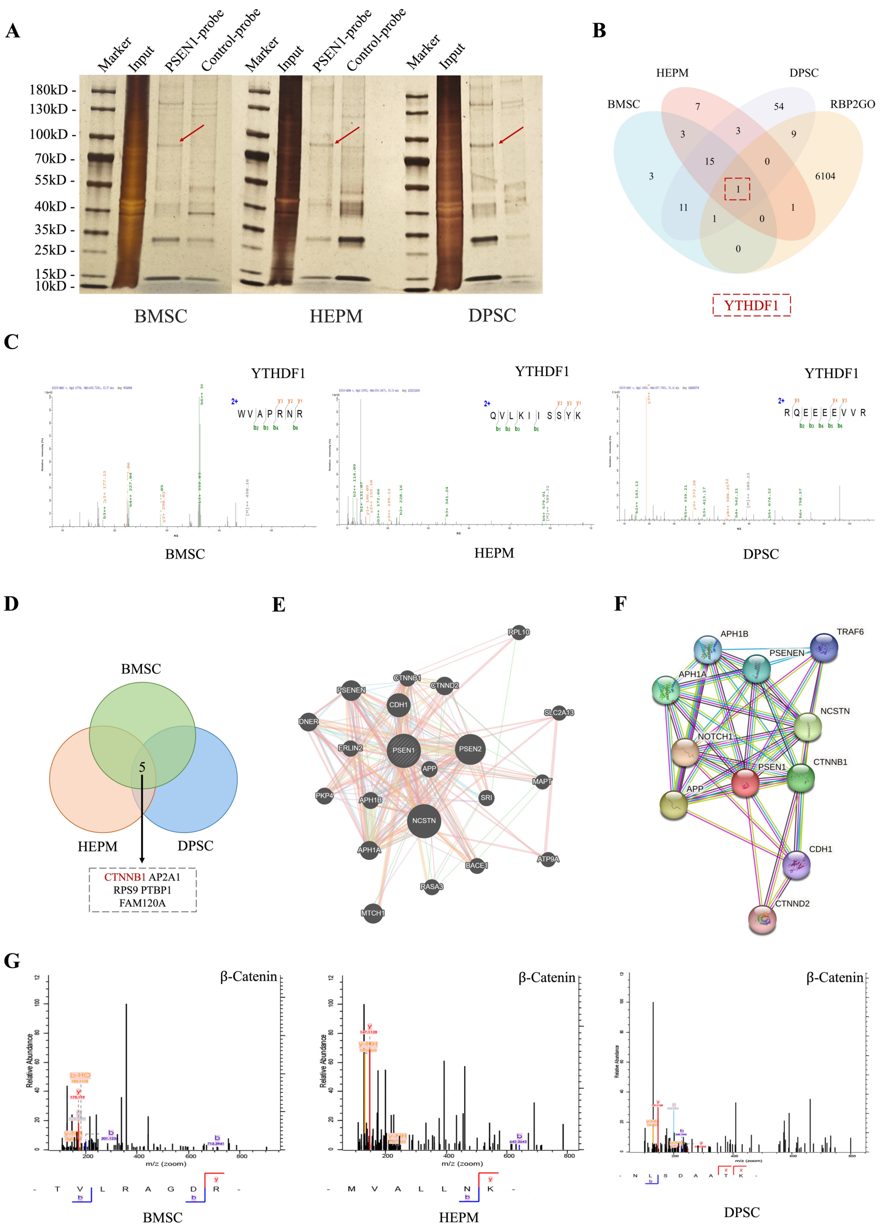

Supplementary Fig. 7

(**A**) The RNA binding proteins determined by silver staining. (**B**) The Venn diagram of RNA binding proteins identified by mass spectrometry (MS) analysis of the corresponding bands in BMSCs, HEPM cells and DPSCs. (**C**) The MS^2 spectrum of YTHDF1 in the three cells. (**D**) The co-IP coupled with MS analysis showing five proteins binding PSEN1 in BMSCs, HEPM cells and DPSCs as illustrated in a Venn diagram. (**E** and **F**) PSEN1 was predicted to bind with CTNNB1 by STRING and GEMANIA. (**G**) The MS^2 spectrum of β-catenin in the three cells.

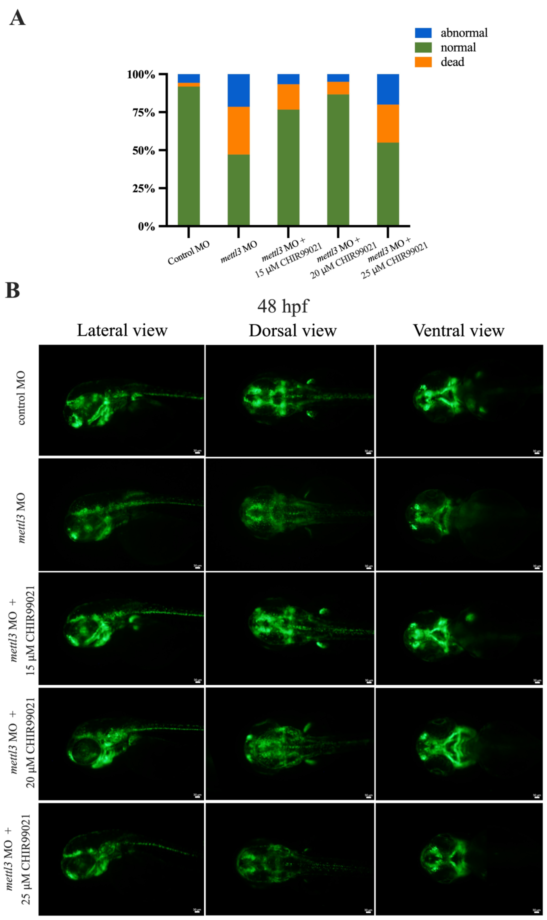

Supplementary Fig. 8

(**A**) Statistical analysis of the number of dead, abnormal or normal embryos at concentrations of 15, 20 or 25 μM CHIR99021. (**B**) The effects of CHIR99021 at different doses on *Tg*(*sox10: eGFP*) transgenic zebrafish embryos at 48 hpf.

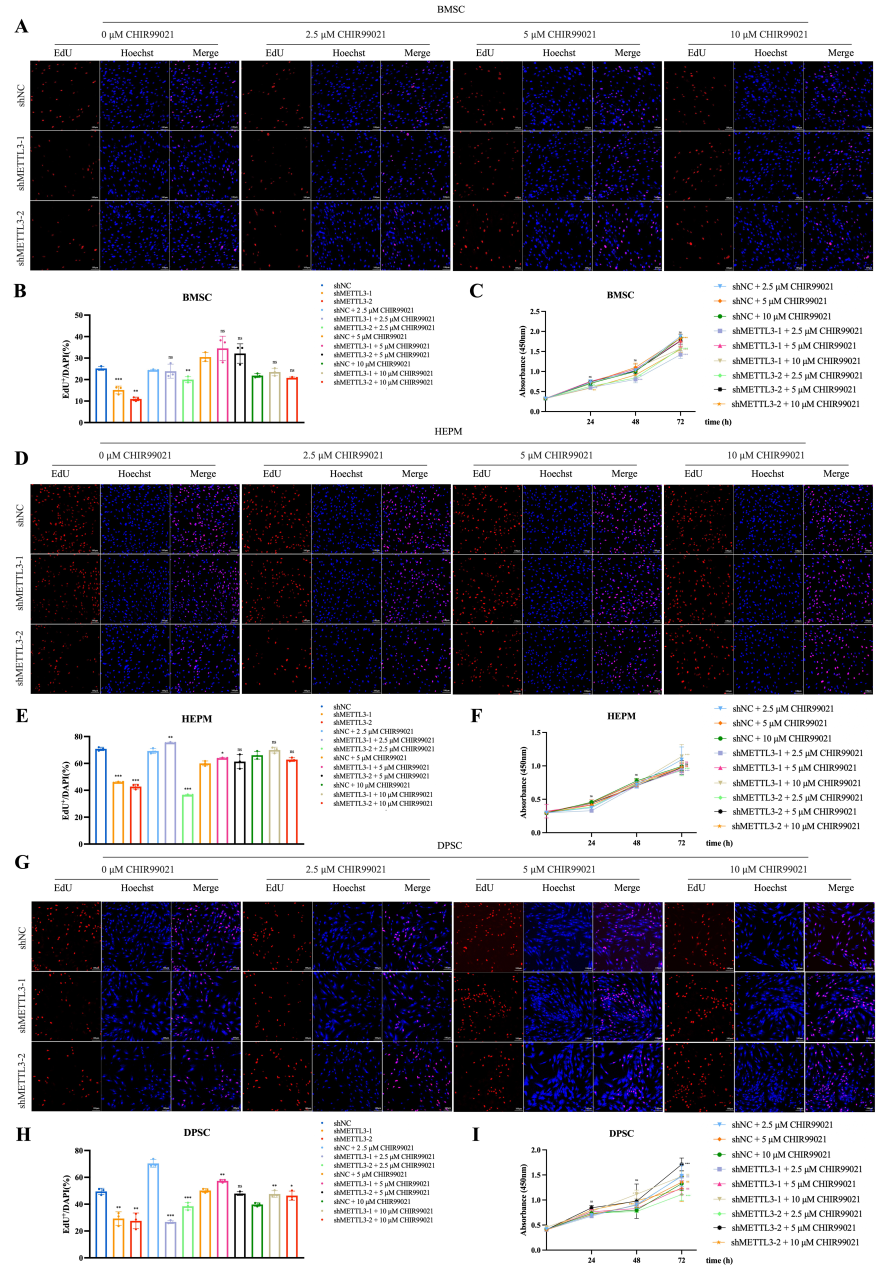

Supplementary Fig. 9

(**A** to **C**) The effects of CHIR99021 at different doses on METTL3-silenced BMSC cell proliferation determined by EdU and CCK8 assays. (**D** to **F**) The effects of CHIR99021 at different doses on METTL3-silenced HEPM cell proliferation determined by EdU and CCK8 assays. (**G** to **I**) The effects of CHIR99021 at different doses on METTL3-silenced DPSC cell proliferation determined by EdU and CCK8 assays.

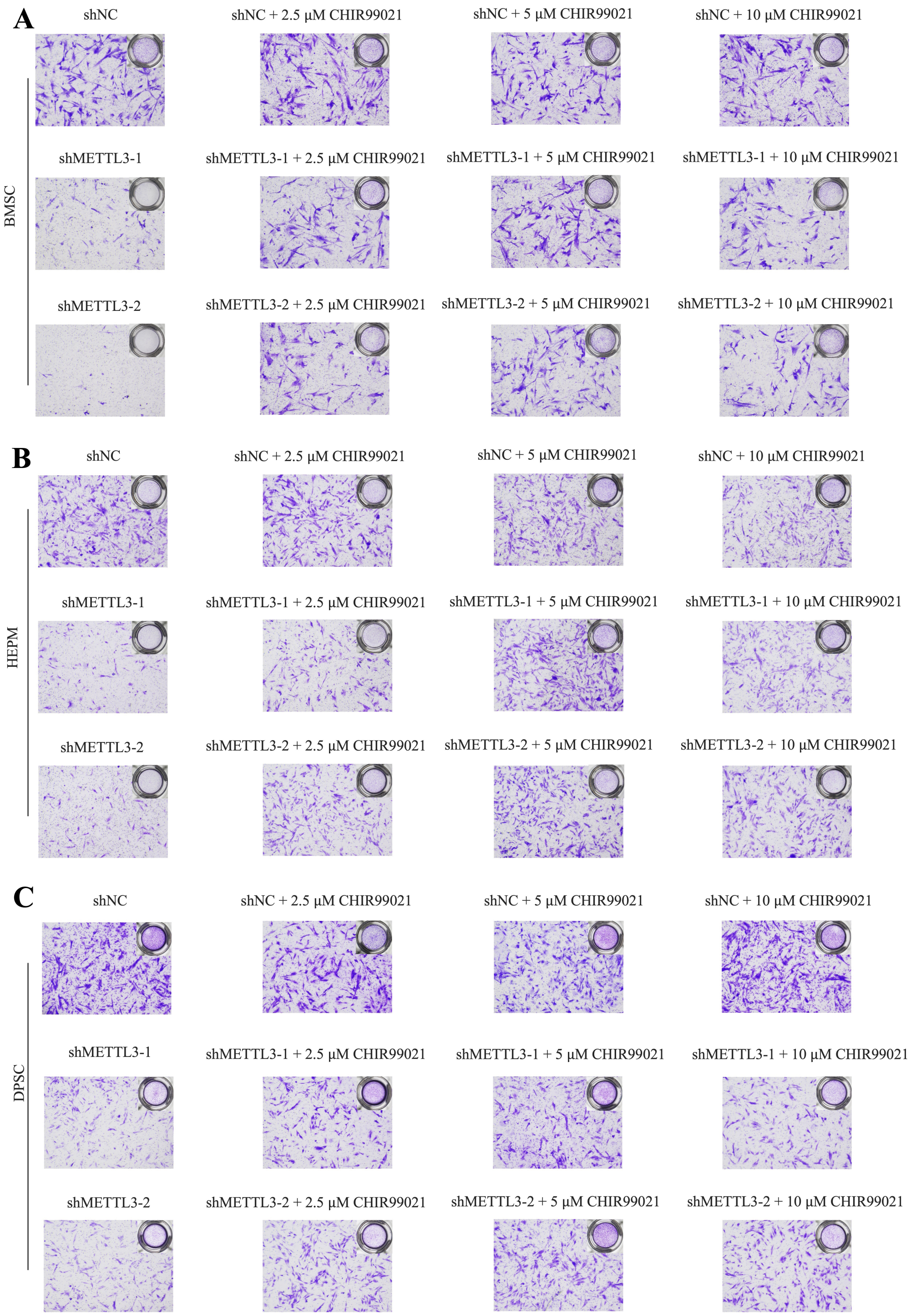

Supplementary Fig. 10

(**A** to **C**) The effects of CHIR99021 at different doses on the migration abilities in METTL3-silenced BMSCs, HEPM cells and DPSCs determined by a transwell assay.

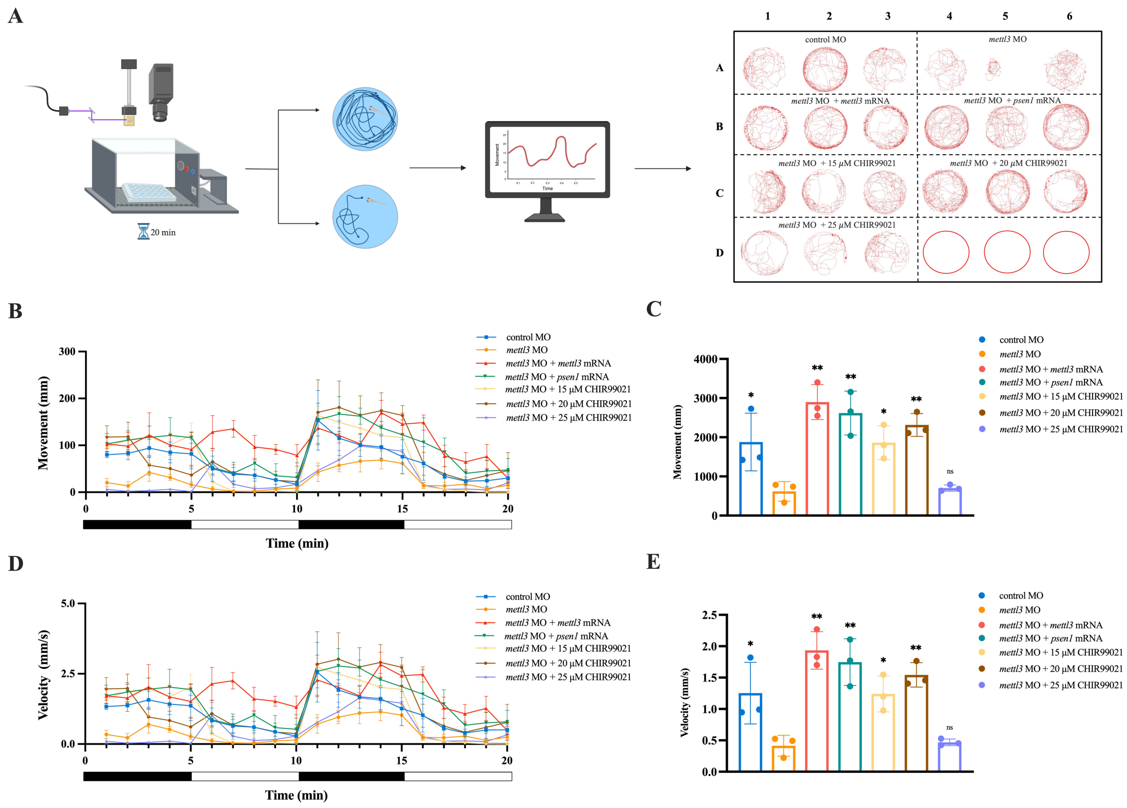

Supplementary Fig. 11

(**A**) A system of behavioral experiments for recording larval motion trials in 20 min, and three representative photographs are shown for each group. (**B**) Swimming distances were binned into 1-min intervals for analysis. (**C**) The total movement of zebrafish larvae in each treatment group at 144 hpf was examined during the dark-light photoperiod stimulation test (20 min). (**D**) The velocity behavioral data were binned into 1-min intervals for analysis. (**E**) The average velocity of zebrafish larvae in each treatment group at 144 hpf was examined during the dark-light photoperiod stimulation test (20 min). Statistics were calculated by unpaired two-tailed Student's *t*-test.
